# Sonic hedgehog is basolaterally sorted from the TGN and transcytosed to the apical domain involving Dispatched-1 at Rab11-ARE

**DOI:** 10.3389/fcell.2022.833175

**Published:** 2022-12-07

**Authors:** Lisette Sandoval, Mariana Labarca, Claudio Retamal, Paula Sánchez, Juan Larraín, Alfonso González

**Affiliations:** ^1^ Centro de Biología Celular y Biomedicina (CEBICEM), Facultad de Medicina y Ciencia, Universidad San Sebastián, Santiago, Chile; ^2^ Centro Ciencia y Vida, Fundación Ciencia para la Vida, Santiago, Chile; ^3^ Centro de Envejecimiento y Regeneración (CARE), Facultad de Ciencias Biológicas, Pontificia Universidad Católica de Chile, Santiago, Chile

**Keywords:** hedgehog, polarity, trafficking, Dispatched, Rab11-ARE, transcytosis

## Abstract

Hedgehog proteins (Hhs) secretion from apical and/or basolateral domains occurs in different epithelial cells impacting development and tissue homeostasis. Palmitoylation and cholesteroylation attach Hhs to membranes, and Dispatched-1 (Disp-1) promotes their release. How these lipidated proteins are handled by the complex secretory and endocytic pathways of polarized epithelial cells remains unknown. We show that polarized Madin–Darby canine kidney cells address newly synthesized sonic hedgehog (Shh) from the TGN to the basolateral cell surface and then to the apical domain through a transcytosis pathway that includes Rab11-apical recycling endosomes (Rab11-ARE). Both palmitoylation and cholesteroylation contribute to this sorting behavior, otherwise Shh lacking these lipid modifications is secreted unpolarized. Disp-1 mediates first basolateral secretion from the TGN and then transcytosis from Rab11-ARE. At the steady state, Shh predominates apically and can be basolaterally transcytosed. This Shh trafficking provides several steps for regulation and variation in different epithelia, subordinating the apical to the basolateral secretion.

## 1 Introduction

Hedgehog proteins (Hhs) are signaling determinants of embryonic patterning, differentiation, and organogenesis, contributing after birth to tissue homeostasis and repair (Beachy et al., 2004; Briscoe and Therond, 2013; Guerrero and Kornberg, 2014; Petrov et al., 2017; Parchure et al., 2018). Alterations of Hh functions are involved in developmental defects, tissue fibrosis, and cancer (Briscoe and Therond, 2013; Edeling et al., 2016; Machado and Diehl, 2017). In most of these processes, the function of Hhs depends on polarized secretion from epithelial cells that possess apical and basolateral domains facing opposite compartments of the organism (Zavros, 2008; Briscoe and Therond, 2013; Guerrero and Kornberg, 2014; Peng et al., 2015; Shyer et al., 2015; Edeling et al., 2016; Petrov et al., 2017; Mao et al., 2018; Walton and Gumucio, 2021). Apical secretion sends signals to neighboring epithelial cells, while basolateral secretion targets neighboring epithelial cells and underlying stromal cells (Chamberlain et al., 2008; Mao et al., 2018; Lehmann et al., 2020; Walton and Gumucio, 2021). Elucidating the Hhs intracellular trafficking pathways in epithelial cells is thus required to fully understand the signaling function of these important morphogens in development, tissue homeostasis, and pathology.

Hhs are synthesized in the endoplasmic reticulum (ER) as a ∼45-kD precursor that is autocatallytically cleaved and processed generating a ∼20-kDa N-terminal peptide, which includes the covalent addition of cholesterol to its C-terminus and palmitate to its N-terminal ends (Porter et al., 1996; Pepinsky et al., 1998; Chen et al., 2011). These lipid moieties firmly attach the mature and functionally competent Hhs to the membrane (Petrov et al., 2017). However, Hhs are released from the plasma membrane by the transmembrane and cholesterol-binding protein Dispatched (Disp) (Burke et al., 1999; Caspary et al., 2002; Creanga et al., 2012; Tukachinsky et al., 2012; Parchure et al., 2018; Stewart et al., 2018). Disp-mediated secretion is driven by the Na^+^ gradient across the plasma membrane and is assisted in mammalian cells by the soluble secreted protein Scube-2 present in the extracellular environment (Creanga et al., 2012; Tukachinsky et al., 2012). The mechanism involves sequential interactions with cholesterol adducts in Hhs, with Disp acting as a cholesterol-Hhs/Na^+^ antiporter followed by Scube-2 as the cholesterol acceptor in the medium (Petrov et al., 2020; Wang et al., 2021). As a member of the RND family of small-molecule transporters, Disp has a prominent extracellular domain (Cannac et al., 2020; Chen et al., 2020), which requires to be cleaved by furin at the plasma membrane (Stewart et al., 2018) to remove a steric impediment to Shh binding (Li et al., 2021).

In different epithelial cells, Hhs have been found localized to the apical, basolateral, or both plasma membrane domains, and their polarized secretion can be inferred from target cell responses. However, the intracellular trafficking pathways of newly synthesized Hhs remain unknown, and this is a limitation to understanding these variations and their functional consequences and potential pathogenic alterations. For instance, Sonic hedgehog (Shh), the most studied vertebrate ortholog of Hhs (Petrov et al., 2017; Parchure et al., 2018), generates paracrine responses of stromal mesenchymal cells indicating basolateral secretion from epithelial cells of adult intestine (Shyer et al., 2015), lungs (Mao et al., 2018), and kidneys (Edeling et al., 2016). Gastric parietal cells mostly distribute and secrete Shh apically, also showing detectable levels at the basolateral side (Zavros et al., 2008). A polarized gastric cell line cultured in permeable membranes secretes Shh both in the apical and basolateral media in response to histamine stimulation (Zavros et al., 2008). In the mature airway epithelia, Shh is also secreted from both apical and basolateral domains eliciting different signaling pathways and functional effects at each compartment (Mao et al., 2018). Shh basolateral release stimulates stromal mesenchymal cells through primary cilia, while its apical secretion induces non-canonical signaling *via* motile cilia (Mao et al., 2018). Shh generated by stromal cells can be internalized through the basolateral cell surface of overlaying epithelial cells and transcytosed to the apical domain for secretion, as shown for Shh produced by the notochorda of mouse embryos, which is basolaterally captured and apically released by ventral midline epithelial cells of the neural tube floor plate (Chamberlain et al., 2008). In the most studied *Drosophila* epithelial systems, Hh distribution, secretion, endocytic recycling, and transcytosis have been described involving apical and basolateral cell surfaces, thus raising debated interpretations of Hh trafficking and signaling routes (Guerrero and Kornberg, 2014; Matusek et al., 2020). In the wing disk epithelia, the proposed models include primary biosynthetic pathway to the apical domain, followed by endocytosis and either apical recycling (D'Angelo et al., 2015) or transcytosis of Hhs to the basolateral domain (Callejo et al., 2011), while in embryonic ectodermal cells, a basolateral primary sorting followed by transcytosis to the apical cell surface has been proposed (Gallet et al., 2003). In all these studies, the Hhs subcellular distributions and secretion have been assessed under steady-state conditions that do not allow to discern the trafficking pathways followed by the newly synthesized Hhs.

The apical and basolateral distribution of specific proteins in epithelial cells depends on sorting events first at the biosynthetic (secretion) route at the TGN level and then at recycling endosomes (Mellman and Nelson, 2008; Golachowska et al., 2010; Rodriguez-Boulan and Macara, 2014). Exocytic pathways emerging from the TGN can directly address the apical or basolateral plasma membrane or include crossroads with recycling endosomes (Ang et al., 2004; Lock and Stow, 2005; Cancino et al., 2007; Cresawn et al., 2007; Gravotta et al., 2007; Gonzalez and Rodriguez-Boulan, 2009; Thuenauer et al., 2014). Once inserted in the corresponding plasma membrane domain, proteins that are endocytosed can recycle back to the same cell surface or follow transcytotic pathways toward the opposite cell surface (Apodaca et al., 1994; Brown et al., 2000; Tuma and Hubbard, 2003; Golachowska et al., 2010). The complex endocytic system of polarized epithelial cells includes separate apical sorting endosomes (ASEs) and basolateral sorting endosomes (BSEs), perinuclear Rab11-negative common recycling endosomes (CREs), and an apical recycling endosome (ARE), which is Rab11-positive and subapically located underneath the primary cilium and over CRE (Sheff et al., 1999; Brown et al., 2000; Folsch et al., 2009; Weisz and Rodriguez-Boulan, 2009; Golachowska et al., 2010). Rab11-ARE integrates a trafficking network that includes apical biogenetic and post-endocytic recycling pathways together with basolateral-to-apical transcytosis routes (Brown et al., 2000; Weisz and Rodriguez-Boulan, 2009; Golachowska et al., 2010; Perez Bay et al., 2016). Protein trafficking along the biosynthetic route and all these endocytic routes is controlled by sorting signals embedded in cargo structures and decoded by a machinery coupled to the generation of vesicular/tubular vehicles (Mellman and Nelson, 2008; Rodriguez-Boulan and Macara, 2014). Transmembrane proteins can bear apical sorting signals in extracellular, membrane-spanning, or cytosolic domains, most of which with little known decoding systems, while basolateral signals are located in cytosolic domains and are mainly decoded by a sorting machinery involving the epithelial-specific AP1B clathrin adaptor (Folsch et al., 1999; Gan et al., 2002) and clathrin (Deborde et al., 2008), probably including also the more general AP1A clathrin adaptor (Gravotta et al., 2012; Guo et al., 2013). Hhs belong to a subgroup of luminal proteins that attach to the membrane through lipid moieties, similar to glycosylphosphatidylinositol-anchored proteins (GPI-APs), which associate with lipid rafts, and their intracellular trafficking routes are comparatively less understood than those of transmembrane proteins (Long et al., 2015; Petrov et al., 2017; Parchure et al., 2018). Lipid rafts have long been considered platforms for apical trafficking (Simons and Ikonen, 1997), but raft-associated proteins can also be found in the basolateral plasma membrane (Imjeti et al., 2011; Paladino et al., 2015; Sezgin et al., 2017; Lebreton et al., 2019; Lebreton et al., 2021).

Madin–Darby canine kidney (MDCK) cells forming polarized monolayers in culture constitute the most characterized model system for studies of protein trafficking in epithelial cells (Rodriguez-Boulan and Macara, 2014). In these cells, secretory proteins lacking polarity sorting signals are distributed unpolarized reflecting TGN-derived apical and basolateral routes of equivalent capacity (Gottlieb et al., 1986; Gonzalez et al., 1987). Therefore, domain-selective secretion implies specific sorting events occurring at the TGN, together or not with additional sorting at transendosomal and/or transcytosis routes in these cells (Mellman and Nelson, 2008; Gonzalez and Rodriguez-Boulan, 2009; Rodriguez-Boulan and Macara, 2014). A unique study of Shh expression in transfected MDCK cells describes Shh apical and basolateral distribution and enhanced basolateral secretion when Disp-1 is coexpressed (Etheridge et al., 2010). However, this study did not assess the apical secretion and as other studies under steady-state conditions leaves uncertain the Shh biosynthetic trafficking.

Here, we used polarized MDCK cells and provided evidence from pulse chase, antibody-tagging, and live-cell imaging analyses that newly synthesized Shh is sorted from the TGN to the basolateral cell surface and then becomes transcytosed to the apical cell surface, from where most of its secretion occurs, while a small proportion returns to the basolateral domain. This Shh polarized sorting requires membrane attachment through its lipid adducts. Colocalization with Rab11 in a subapical structure suggests that Shh follows the basolateral-to-apical transcytosis route involving the Rab11-ARE compartment. Microinjection experiments to coexpress Shh together with Disp-1 or a secretion-defective Disp-1 mutant reveal that Disp-1 promotes post-TGN basolateral secretion of newly synthesized Shh and then participates in its transcytosis pathway at the Rab11-ARE stage.

## 2 Results

### 2.1 Predominant apical distribution and secretion of Sonic hedgehog in polarized epithelial MDCK cells

**FIGURE 1:**
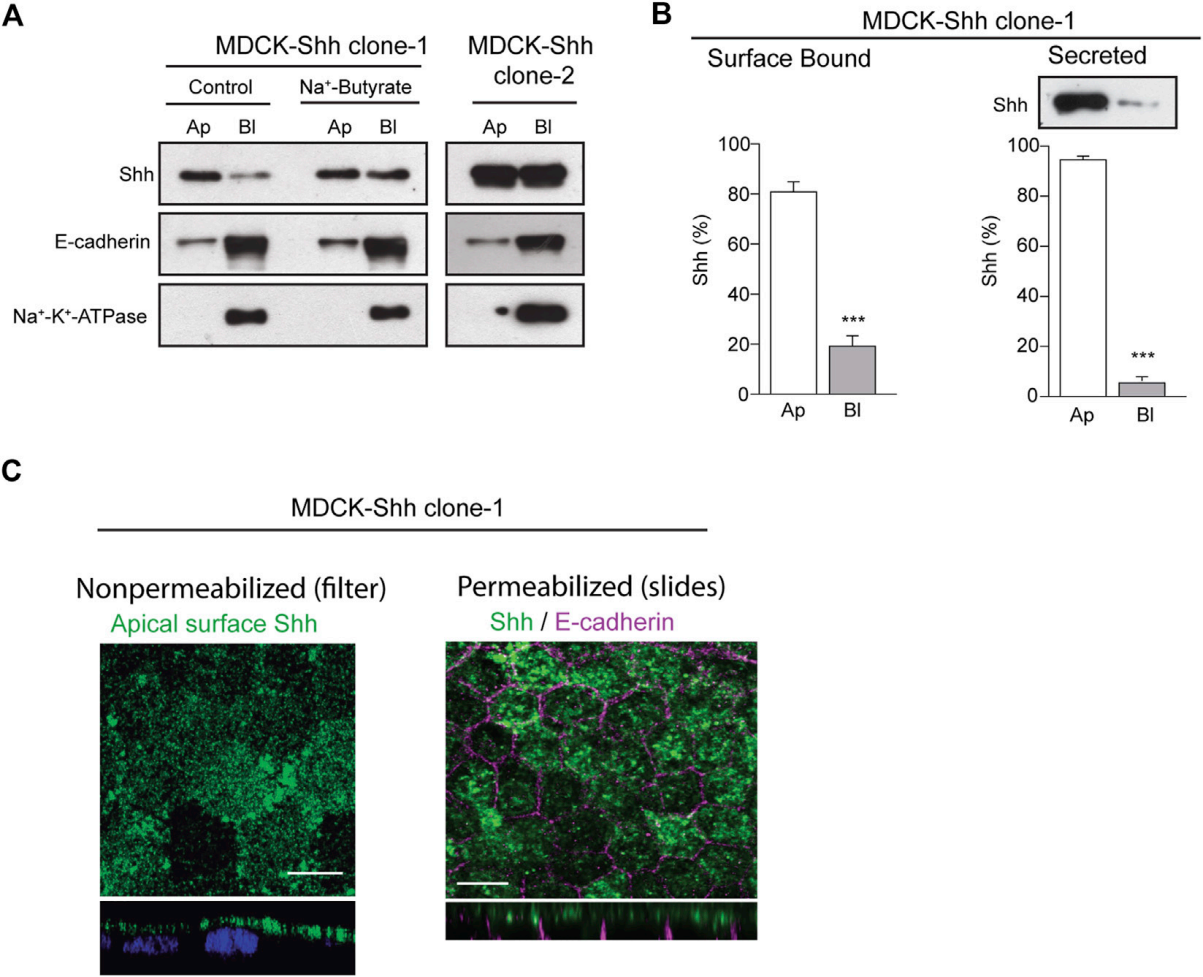
Shh is apically distributed and secreted in stable transfected MDCK cells. **(A)** Domain-selective biotinylation of stable transfected MDCK-Shh grown in Transwell filters. MDCK-Shh clone-1 shows Shh mainly at the apical cell surface, changing to non-polarized when cells are treated with 2 mM Na + -butyrate for 24 h to increase Shh expression. MDCK-Shh clone-2 expressing higher Shh levels than clone-1 displays Shh equally distributed among the apical and basolateral cell surface. E-cadherin and Na^+^/K^+^ -ATPase basolateral distribution corroborates the polarized status of the cells. **(B)** Graphs show ∼80% apical cell surface distribution (n = 6) and more than 90% apical secretion of Shh in 6 h conditioned media in MDCK-Shh clone-1 (n = 3), (***p < 0.001); **(C)** Polarized MDCK-Shh clone 1 cells immunostained for apical cell surface Shh in non-permeabilized cells grown in filters, as well as for total Shh (green) and E-cadherin (magenta) in cells grown in glass coverslips, as indicated. Images are confocal z-stacks maximum projections. Scale bar, 10 μm.

To analyze the distribution of Shh in the context of epithelial cell polarity, we generated stably transfected MDCK cells expressing different levels of Shh and first assessed the apical/basolateral distribution and secretion under steady-state conditions. Domain-specific cell surface biotinylation of polarized MDCK-Shh cells grown in Transwell chambers showed cell clones with Shh predominantly apical, like MDCK-Shh clone-1, whereas in other clones, such as MDCK-Shh clone-2, Shh is unpolarized seemingly due to higher expression levels (Figure 1A). E-cadherin and Na^+^-K^+^-ATPase, two basolateral proteins, demonstrated that both clones address other proteins with the expected polarity (Figure 1A). To confirm that high transfected-Shh expression levels lead to polarity loss, we treated MDCK-Shh clone 1 with Na^+^-butyrate, which enhances the expression of transfected plasmids (Gonzalez et al., 1987; Burgos et al., 2004; Thompson et al., 2007). Shh is distributed now with similar apical and basolateral levels, while endogenous E-cadherin and Na^+^-K^+^-ATPase maintained their basolateral polarity (Figure 1A). Quantitative assessment of Shh cell surface distribution and secretion in MDCK-Shh clone-1, named from now on as MDCK-Shh, showed Shh ∼80% at the apical cell surface and more than 90% apically secreted (Figure 1B). These results indicate that Shh is predominantly sorted to the apical cell surface from where most of its secretion occurs and suggest a sorting process that can be saturated over certain expression levels.

To detect Shh at the apical cell surface by immunofluorescence, we added anti-Shh primary antibody to the apical pole of polarized MDCK-Shh grown in filter chambers (Figure 1C, left panel). A similar apical staining pattern was obtained in polarized MDCK cells grown in glass coverslips, fixed, and permeabilized 3–4 days after reaching confluence, contrasting with the basolateral marker E-cadherin (Figure 1C, right panel). We have shown that under these growing conditions, the MDCK cells develop primary cilia (Donoso et al., 2009), and here we add that they also acquire a subapical punctate-like distribution of Rab11a (see below in Figure 5C), both features considered hallmarks of polarity development (Casanova et al., 1999; Brown et al., 2000; Folsch et al., 2009; Perez Bay et al., 2013).

### 2.2 Shh is basolaterally sorted and then transcytosed to the apical cell surface

**FIGURE 2:**
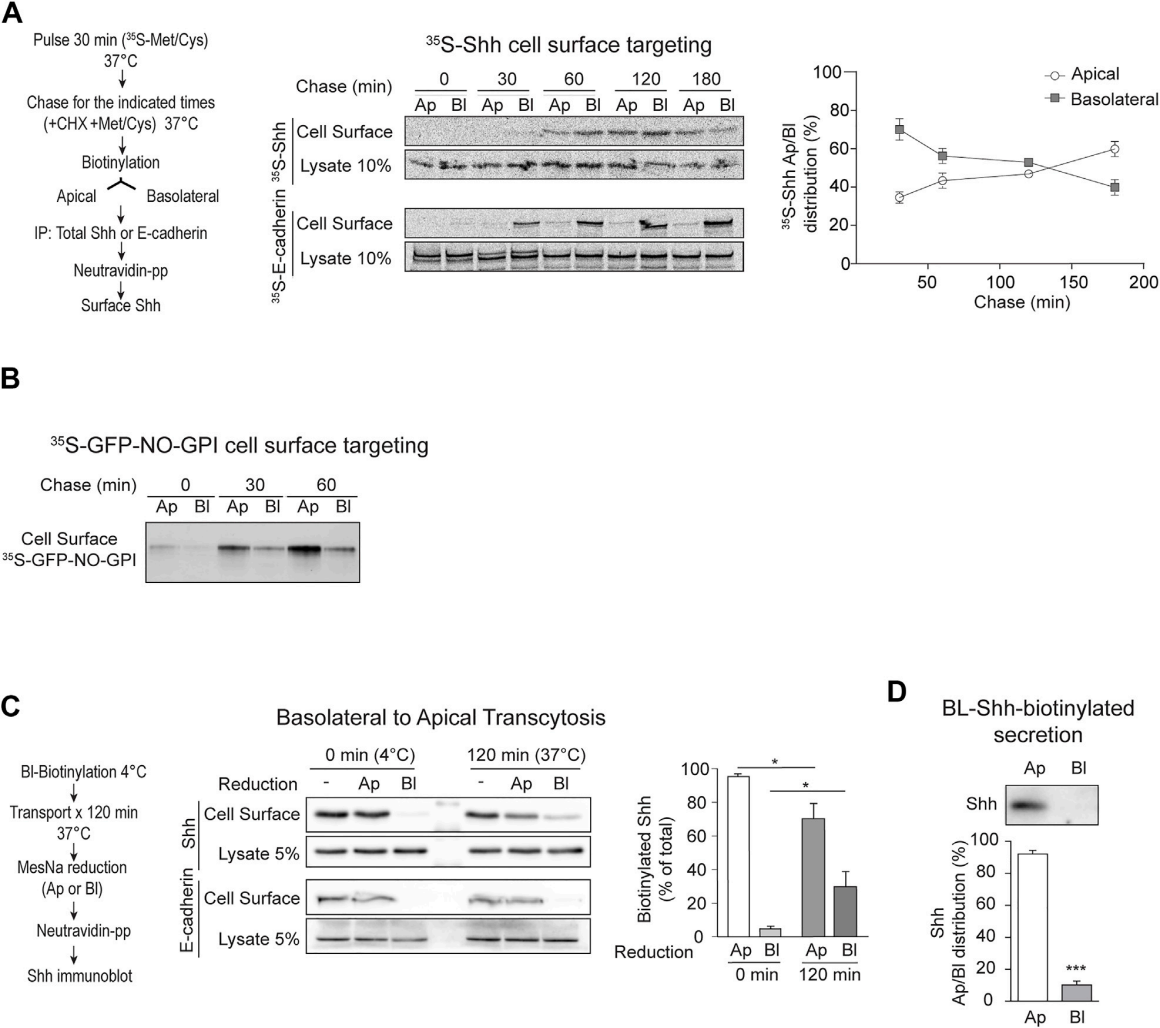
Apical sorting of newly synthesized Shh is indirect through transcytosis. **(A)** Cell surface targeting assay of newly synthesized Shh and E-cadherin in polarized MDCK-Shh cells grown in Transwell filters. The cells were pulse-labeled with ^35^S-methionine/cysteine and chased at 37°C for the indicated times by subsequent cell-surface-specific biotinylation at 4°C. S^35^-Shh is first detected at the basolateral cell surface and then gradually increased at the apical domain, while S^35^-E-cadherin remains basolateral. Graph shows the relative distribution of S^35^-Shh at different time points (30′, n = 5; 60′, n = 7; 120′, n = 4; and 180′, n = 2). **(B)** Cell surface targeting assay of newly synthesized GPI-anchored protein (GFP-NO-GPI) shows direct apical sorting. **(C)** Shh basolateral-to-apical transcytosis assay. Filter-grown MDCK-Shh cells were basolaterally labeled at 4°C with reducible biotin linkage (NHS-SS-Biotin), incubated at 37°C for 120 min and then subjected to MesNa reduction at apical or basolateral sides. Shh-biotin, but not E-cadherin–biotin, decreased by reduction at the apical side indicating Shh transcytosis. Graph shows percentage of Shh-biotin after reduction (n = 3; mean ± SEM; unpaired *t*-test; *p < 0.05). **(D)** Basolateral cell surface Shh becomes apically secreted. MDCK-Shh cells biotinylated from the basolateral side at 4°C and then incubated at 37°C for 2 h mainly release biotinylated Shh toward the apical media. Graph shows the percentage of total secreted biotinylated Shh precipitated with NeutrAvidin–Agarose beads followed by immunoblot (n = 4; mean ± SEM; unpaired *t*-test; ***p < 0.001).

To test whether newly synthesized Shh is directly sorted to the apical cell surface or follows an indirect basolateral-to-apical transcytotic route, we pulse-labeled MDCK-Shh cells with [^35^S]-methionine/cysteine and then chased the arrival of newly synthesized [^35^S]-Shh to the apical and basolateral cell surfaces using an established biotinylation targeting assay (Le Bivic et al., 1989; Zurzolo et al., 1992). [^35^S]-Shh first appeared at the basolateral surface and then progressively increased at the apical cell surface, whereas E-cadherin displayed basolateral distribution at all chased times (Figure 2A). To test whether this targeting assay is able to detect direct apical sorting, we used a previously characterized recombinant GPI-AP model protein, named GFP-NO-GPI (Imjeti et al., 2011). This protein contains N- and O-glycosylation and associates with lipid rafts, two features involved in direct apical sorting from the TGN (Hua et al., 2006; Paladino et al., 2006; Imjeti et al., 2011). In contrast with Shh, GFP-NO-GPI reached the apical cell surface within 30–60 min of chasing time and did not show a previous basolateral distribution (Figure 2B), indicating direct apical sorting. These results suggest that newly synthesized Shh is primarily sorted to the basolateral domain in the biosynthetic route and then becomes transported by transcytosis to the apical cell surface.

Next, we directly assessed basolateral-to-apical transcytosis with an established assay using the reducible reagent Sulfo-NHS-SS-Biotin (Zurzolo et al., 1992; Burgos et al., 2004). After biotinylation of the basolateral cell surface at 4°C and 120 min of incubating the cells at 37°C to allow transport, we found approximately 25% of biotinylated Shh protected from reduction by MesNa added to the basolateral chamber (Figure 2C). This protection to reduction reflects the endocytosis of Shh from the basolateral cell surface making it inaccessible to MesNa, which does not permeate the membrane. MesNa added to the apical side decreased the levels of biotinylated Shh, but not E-cadherin, thus indicating Shh transcytosis (Figure 2C). Shh biotinylated from the basolateral cell surface was also found in the apical media (Figure 2D). These results directly demonstrate that Shh present at the basolateral cell surface reaches the apical cell surface and is apically secreted by transcytosis.

**FIGURE 3:**
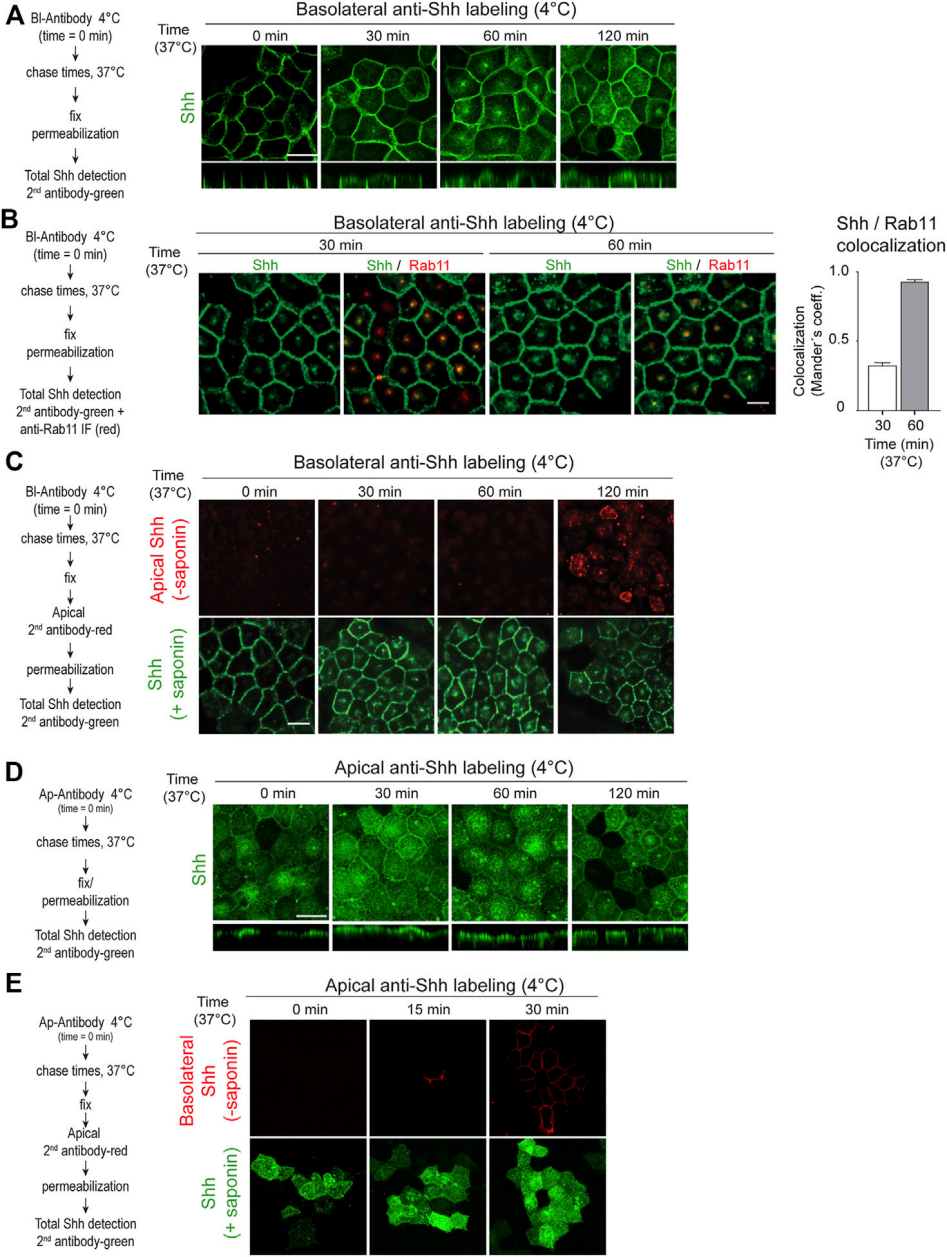
Shh transcytosis. Polarized MDCK-Shh cells in Transwell filters were incubated with anti-Shh antibodies at 4°C from the basolateral **(A**–**C)** or apical **(D**,**E)** sides, shifted to 37°C for the indicated times, and detected by the secondary antibody. **(A)** Antibody-tagged basolateral Shh moved to the apical domain including a subapical punctate compartment, as shown by the secondary antibody (green) added after permeabilizing the cells at the indicated time points. **(B)** Shh (green) colocalization with endogenous Rab11 (red) after 30 and 60 min of trafficking. Graph shows the percentage of Rab11 colocalizing with Shh (mean ± SEM, n = 15 cells, unpaired *t*-test ***p < 0.001. **(C)** Apical cell surface detection of antibody-tagged basolateral Shh. Secondary antibody (red) was added to the apical side of non-permeabilized cells, which then were permeabilized and incubated with another secondary antibody (green). **(D)** Antibody-tagged apical Shh moved to the basolateral domain, as shown by total staining of permeabilized cells. **(E)** Basolateral cell surface detection of antibody-tagged apical Shh. Secondary antibody (red) was added to the basolateral side of non-permeabilized cells, which then were permeabilized and incubated with another secondary antibody (green). Scale bar, 10 μm.

To further corroborate these findings and visualize the transcytosis pathway, we tagged the Shh basolateral pool with anti-Shh antibodies at 4°C, shifted the cells to 37°C for different times, and then followed the distribution of antibody-tagged Shh by immunofluorescence using a secondary antibody. Shh moved from the basolateral cell surface acquiring a dot-like distribution at the subapical region before reaching the apical cell surface (Figure 3A). This particular dot-like structure seems to be the Rab11-ARE compartment (Apodaca et al., 1994; Barroso and Sztul, 1994; Casanova et al., 1999; Wang et al., 2000a; Brown et al., 2000) as judged by its subapical location and colocalization of Rab11 staining with Shh (Figure 3B). Apical addition of the secondary antibody to non-permeabilized cells demonstrated the appearance of basolaterally tagged Shh at the apical cell surface (Figure 3C). Therefore, basolaterally sorted Shh followed a transcytotic route involving the Rab11-ARE compartment.

### 2.3 Shh can recycle back from the apical to the basolateral cell surface

Shh transcytosis from the basolateral to the apical membrane is congruent with one of the models proposed for Hh protein trafficking in *Drosophila* ectoderm epithelia (Gallet et al., 2003; Gallet et al., 2006). However, the opposite apical-to-basolateral Hh transcytosis has been proposed in *Drosophila* wing disk epithelial cells (Callejo et al., 2011; Guerrero and Kornberg, 2014). Our antibody-tagging assay showed that Shh present at the apical cell surface can also be transported to the basolateral cell surface (Figures 3D,E). Therefore, even though newly synthesized Shh once arriving to the apical surface is mostly secreted to the apical media (See Figures 1, 2), a small proportion can recycle back to the basolateral cell surface.

### 2.4 Role of lipid modifications in Shh polarized sorting

**FIGURE 4:**
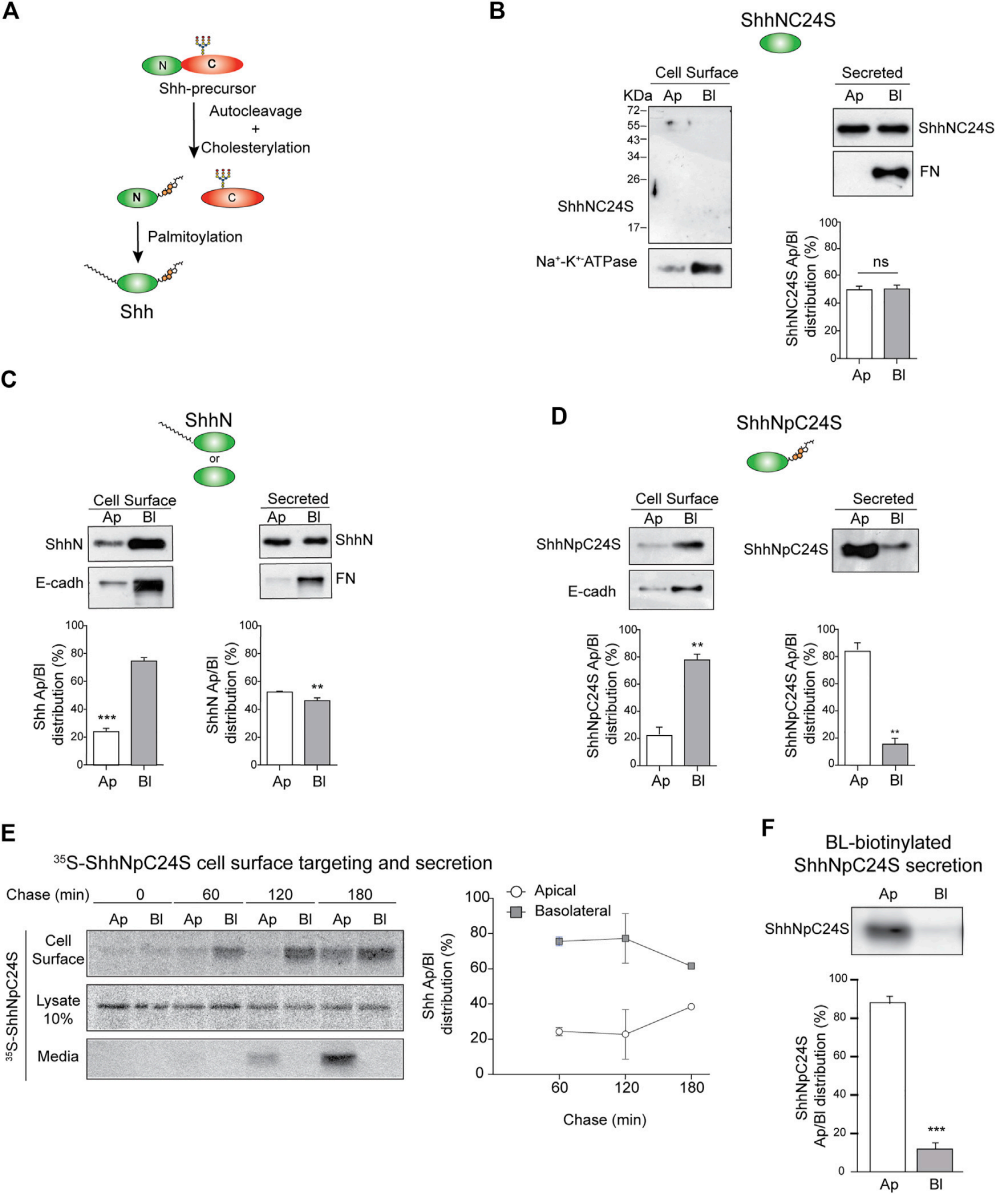
Sorting of Shh lipidation mutants. **(A)** Schematic Shh processing and lipidation. **(B**–**F)** Stable expression of MDCK cells indicated Shh lipidation mutants: lacking both lipids (ShhNC24S) **(B)**, only palmitoylated (ShhN) **(C)**, or only cholesteroylated (ShhNpC24S) **(D)** were analyzed by domain-specific cell surface biotinylation and secretion. Basolateral E-cadherin and Na^+^/K^+^ ATPase transmembrane proteins and secreted fibronectin (FN) show cell polarity. **(E)** MDCK-ShhNpC24S cells were pulse-labeled with S^35^-methionine/cysteine and chased to detect protein arrival to each cell surface along with its polarized secretion. Graph shows the mean±SEM from two independent experiments. **(F)** Secretion of transcytosed ShhNpC24S. MDCK-ShhNpC24S cells biotinylated from the basolateral side at 4°C were then incubated at 37°C for 2 h, and immunoblot of secreted ShhNpC24S was performed on apical and basolateral conditioned media. Graphs of biotinylation and secretion assays (n = 3) represent mean±SEM. **p < 0.05; ***p < 0.001, ns = no significance.

Hhs are synthesized as precursor proteins that are autocleaved and processed by cholesteroylation at the C-terminus and palmitoylation at the N-terminus (Porter et al., 1996; Pepinsky et al., 1998; Chen et al., 2011) (Figure 4A). These lipid modifications determine Hh membrane association, nano-scale organization, secretion mode, and action range (Petrov et al., 2017; Parchure et al., 2018). We tested the role of lipidation in Shh polarized sorting using mutants lacking both palmitoylation and cholesteroylation (ShhNC24S), lacking only cholesteroylation (ShhN), or lacking only palmitoylation (ShhNpC24S). The unlipidated ShhNC24S mutant was equally secreted to both the apical and basolateral media (Figure 4B). Without cholesteroylation, ShhN was found associated to the basolateral cell surface and secreted unpolarized (Figure 4C). In contrast, ShhNpC24S lacking the cysteine for palmitoylation and conserving the cholesterol modification preferentially distributed to the basolateral membrane and was mostly apically secreted (Figure 4D). Pulse-chase assays showed that the newly synthesized ShhNpC24S arrives first at the basolateral cell surface and then at the apical cell surface and medium (Figure 4E), mimicking the pathway of normal lipidated Shh (See Figure 2A). However, ShhNpC24S achieved low levels at the apical cell surface suggesting that it is less sustained at this membrane and thus easily released to the media. ShhNpC24S became detectable at the apical media after 120 min of chase, while most of its membrane-associated form is seen at the basolateral cell surface (Figure 4E, middle). We corroborated an apical secretion of basolaterally biotinylated-ShhNpC24S (Figure 4F). All these observations reveal that both palmitoyl and cholesterol modifications contribute to Shh sorting to the basolateral cell surface. At least the cholesterol adduct would be further required for subsequent Shh transcytosis.

### 2.5 Disp-1 distribution and role in Shh secretion

**FIGURE 5:**
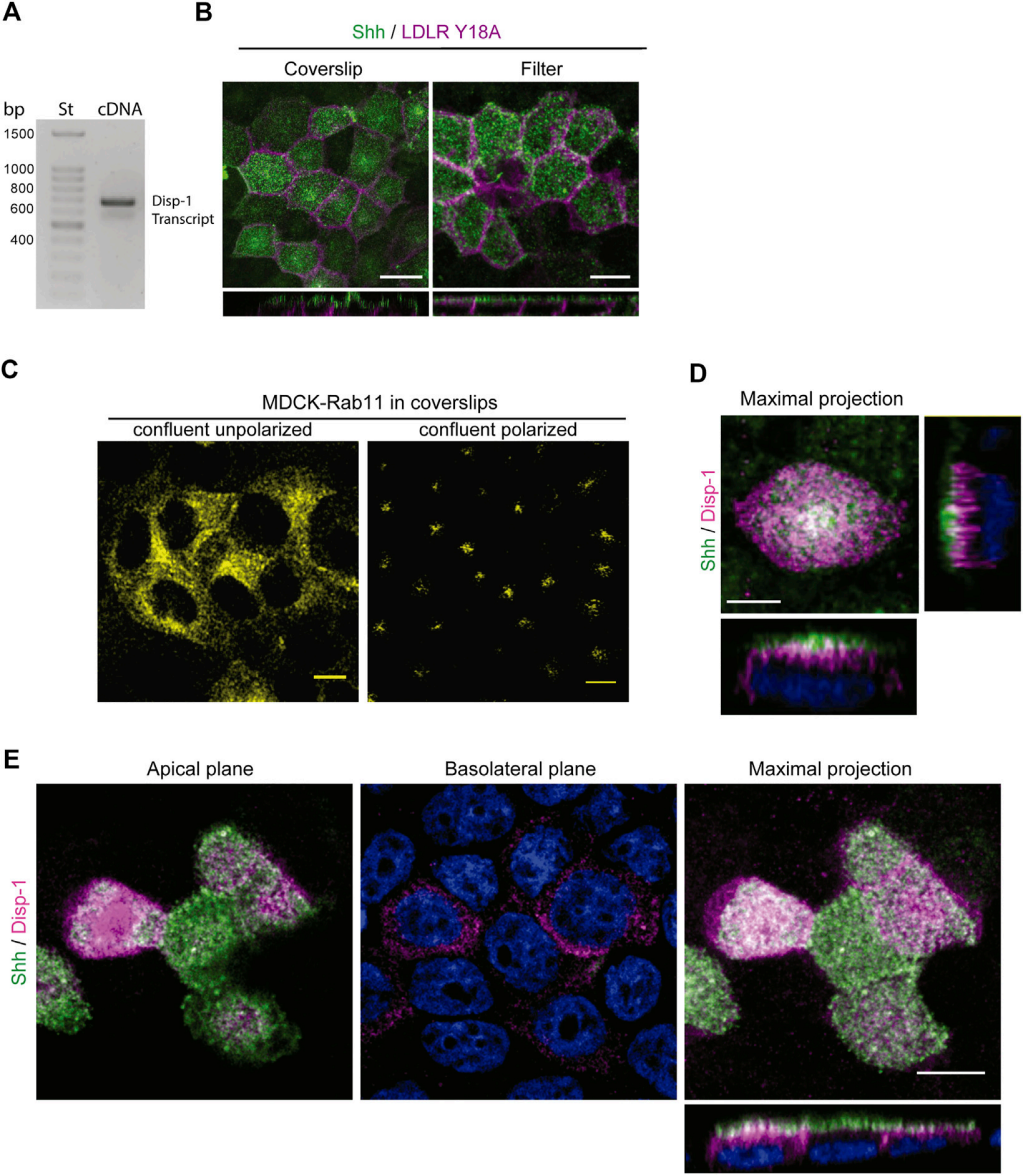
Distribution of Disp-1 in polarized MDCK cells. **(A)** Endogenous Disp-1 expression in MDCK cells. RT-PCR shows the expected band of 562 bp according to primers (see Materials and Methods). **(B)** MDCK cells grown in glass coverslips or Transwell filters for 3–4 days after reaching confluence were microinjected with expression plasmids for Shh and LDLR-GFP-Y18A, incubated for 4 h at 37°C and processed to detect each expressed protein. Indirect immunofluorescence of Shh (green) and direct imaging of LDLR-GFP-Y18A (magenta) in permeabilized cells show a similar apical and basolateral distribution, respectively, in glass coverslips and filters, validating the polarity conditions. **(C)** Rab11-ARE distribution in non-polarized and polarized MDCK cells. MDCK cells stably transfected with Rab11-CFP cDNA were grown on glass coverslips until confluence (left) or 3–4 days after confluence (right), then fixed, and imaged by confocal microscopy. The characteristic disperse perinuclear distribution is shown in non-polarized cells, whereas the punctate-like subapical localization is observed after 3–4 days of confluence in coverslips. **(D)** Distribution of Shh relative to Disp-1 expressed by plasmid microinjection. Polarized MDCK cells in glass coverslips were microinjected to coexpress Shh and Disp-1, fixed and immuno-stained by the apical side with anti-Shh (green) antibody, and then permeabilized and stained for Disp-1 with anti-HA (magenta) antibodies. Disp-1 mainly distributes underneath the apical cell surface stained by non-permeabilized anti-Shh antiody. **(E)** Polarized MDCK cells grown and treated as in **(D)**, were fixed, permeabilized, and stained for total anti-Shh (green) and anti-HA (magenta), showing similar Disp-1 and Shh distribution as in **(D)**. Scale bar, 10 μm.

The release of Hhs from the plasma membrane involves Disp function (Creanga et al., 2012; Tukachinsky et al., 2012; Cannac et al., 2020; Chen et al., 2020; Petrov et al., 2020; Wang et al., 2021). In *Drosophila* epithelial cells, Disp has been shown to promote Hh secretion from both the apical and basolateral cell surfaces in correlation with its detection at both domains, including regionalized endosomal compartments (Etheridge et al., 2010; Callejo et al., 2011; Stewart et al., 2018). Overexpression by transient transfection experiments in MDCK cells has shown that Disp-1 stimulates Shh basolateral release (Etheridge et al., 2010). These studies do not evaluate the role of Disp on newly synthesized Hhs, and the overexpression conditions may distort the trafficking pathways as we showed here for Shh (see Figure 1A). We found by RT-PCR analysis that MDCK cells endogenously express Disp-1 (Figure 5A). However, we could not define its subcellular distribution because there is not a suitable antibody and our attempts to obtain MDCK cells stably transfected with an epitope-tagged Disp-1 (Stewart et al., 2018) failed. MDCK cells seemingly do not tolerate prolonged periods of high Disp-1 expression levels. Therefore, to assess the role of Disp in Shh distribution and secretion, we used plasmid microinjection experiments, which allow to achieve detectable levels of expression for short periods of time, minimizing compensatory effects and missorting due to saturation (Kreitzer et al., 2003; Cancino et al., 2007). Microinjection experiments are more easily and conveniently performed in cells grown in glass coverslips where polarity features can be achieved after 3–4 days of confluence (Jaulin et al., 2007; Guo et al., 2013; Perez Bay et al., 2013). Microinjected polarized MDCK cells grown in filters or glass coverslips similarly displayed the expected polarized distributions of co-expressed Shh (apical) and LDLR^Y18A^-GFP basolateral marker (Figure 5B). Also, the subapical distribution of Rab11-ARE was similarly achieved regardless of the supporting substrate, as shown in Figure 3B (filters) and 6B (coverslips). Figure 5C shows that non-polarized confluent MDCK cells exhibit a perinuclear disperse location of Rab11, changing to subapical dense distribution after 3–4 days of confluence in glass coverslips, as described for fully polarized MDCK cells (Casanova et al., 1999; Brown et al., 2000; Perez Bay et al., 2013). Therefore, we performed further experiments on polarized MDCK cells grown in glass coverslips, expressing Shh alone or together with Disp-1 tagged in its intracellular domain with an HA epitope (Stewart et al., 2018). Contrasting with the main basolateral distribution described in transiently transfected MDCK cells (Etheridge et al., 2010), plasmid microinjection coexpressed Disp-1 mainly localized at the apical region, just underneath the cell surface located Shh, showing few spots of colocalization between both proteins (Figure 5D). For this experiment, we first added anti-Shh antibody to intact cells, thus staining Shh at the apical cell surface, and then permeabilized the cells and added the anti-HA antibody to detect Disp. Similar images were obtained by simultaneously detecting total Shh and Disp-1 in permeabilized cells (Figure 5E). Separate apical and basolateral confocal planes further demonstrated the main apical distribution of Shh and Disp-1, detecting also a slight staining of both proteins at the basolateral border (Figures 5D,E Basolateral plane).

**FIGURE 6:**
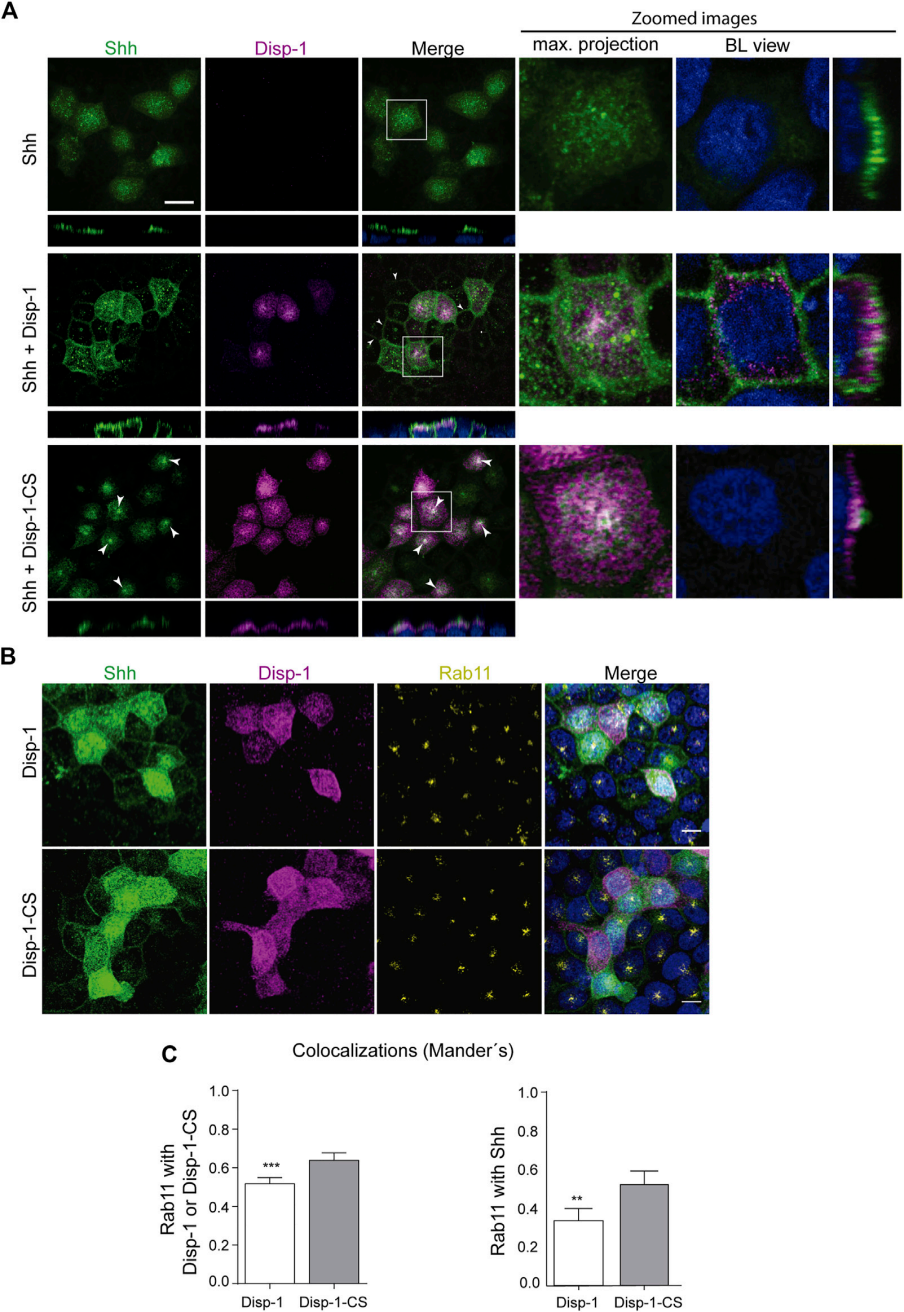
Distribution of newly synthesized Shh when co-expressed with Disp-1 or Disp-1-CS. **(A)** Polarized MDCK cells in glass coverslips were microinjected to express Shh alone (upper panels), together with Disp-1 (middle panels), or Disp-1-CS (lower panels). Images of indirect immunofluorescence for Shh (green) and Disp-1 proteins (HA, magenta) are shown in maximal projections with zoomed images including basolateral (BL) views at the right. When coexpressed with Disp-1, Shh is not only distributed at the apical domain but also appears at basolateral borders of non-microinjected neighboring cells (arrow heads), reflecting its increased secretion. Meanwhile, when coexpressed with Disp-1-CS, Shh became enriched in a punctate subapical compartment (arrow heads) reminiscent of Rab11-ARE. **(B)** Colocalization of Rab11 with Shh and Disp-1. Polarized MDCK cells in glass coverslips were microinjected with Shh and Disp-1 plasmids, allowed for expression for 4 h at 37°C, fixed, permeabilized, and immunostained for Shh (green), Disp-1 or Disp-1-CS (magenta), and endogenous Rab11 (yellow). **(C)** Graphs quantified Rab11 colocalization with each protein in cells coexpressing either Disp-1 or Disp-1-CS, as indicated. Rab11 colocalized more with coexpressed Disp-1-CS than Disp-1 (left graph). Co-expressed Disp-1-CS increased Rab11 colocalization with Shh (right graph) (mean±SEM, n = 25 cells from at least three different experiments). ***p < 0.001. Scale bar, 10 μm.

The following experiments compared the distribution of Shh expressed alone and together with either Disp-1 or the cleavage-deficient and secretion-incompetent Disp-1-CS mutant (Stewart et al., 2018). As before, both Shh expressed alone or together with Disp-1 showed predominant apical distribution (Figure 6A, upper and middle panels). Disp-1 occupied a subapical location overlaid by Shh, again with few spots of colocalization (Figure 6A, middle panel). Shh became clearly visible at the basolateral border of non-microinjected cells that are adjacent to Shh and Disp-1 coexpressing cells, thus indicating an enhanced Shh basolateral secretion (arrow heads in Figure 6A, middle panel). These images are similar to those described in Etheridge et al. (2010) in MDCK transiently cotransfected cells reflecting an increased basolateral secretion under steady-state conditions. Our results indicate that Disp-1 enhances basolateral secretion of recently synthesized Shh.

**FIGURE 7:**
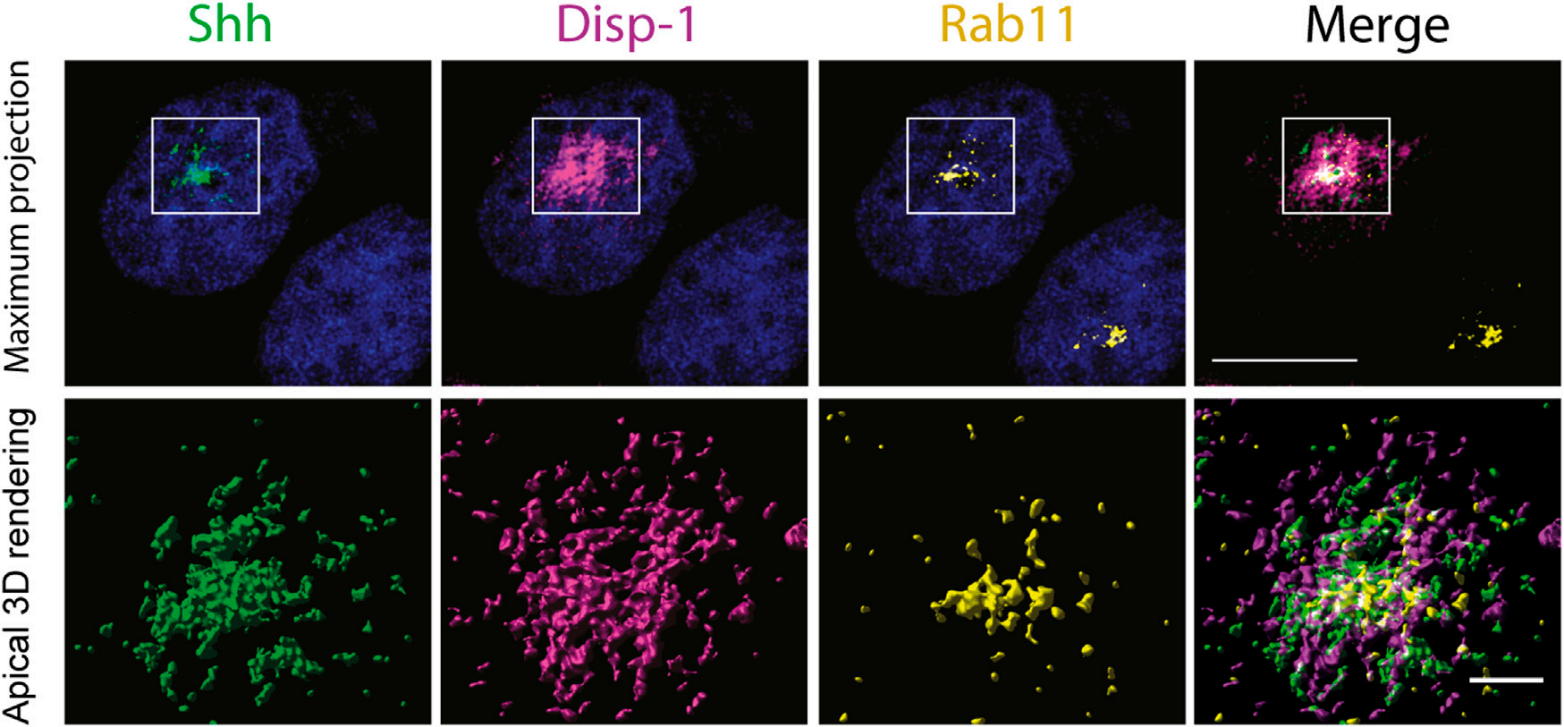
High-resolution image of subapical endosomes containing Shh, Disp-1, and Rab11. Polarized MDCK cells in glass coverslips were microinjected to express Shh and Disp-1 for 4h, fixed, permeabilized, and processed for indirect immunofluorescence of Rab11 (yellow), Shh (green), and Disp-1 (magenta), and 12 confocal images of 100 nm thickness were acquired at the subapical region that include the Rab11 compartment. Deconvolved images are shown as maximum projections (upper panels) and as surface 3D-rendered reconstruction of apical planes (lower panels).

In contrast, cells coexpressing Disp-1-CS did not show the increased Shh basolateral decoration of neighboring cells (Figure 6A, bottom panel), congruent with Disp-1-CS as a secretion-defective mutant (Stewart et al., 2018). Disp-1-CS coexpressing cells frequently showed higher Shh staining at the subapical compartment reminiscent of Rab11-ARE (Figure 6A, bottom panel). To corroborate that this punctate structure corresponds to Rab11-ARE, we performed a similar experiment evaluating Rab11 immunofluorescence (Figure 6B) and colocalization (Figure 6C). Rab11 not only colocalized with Shh and Disp-1 in the subapical compartment but also showed higher colocalization with Shh in cells coexpressing Disp-1-CS (Figure 6C). To have a more detailed image, we performed high-resolution analysis (3D surface rendering) of the subapical region (Figure 7). The presence of Shh and Disp-1 was more clearly seen in subapical vesicular and tubular structures, which most likely include apical sorting endosomes in addition to Rab11-ARE (Figure 7). All these results indicate that Disp-1 but not Disp-1-CS promotes the release of newly synthesized Shh from the basolateral surface where it first arrived and suggest that Disp-1 also has a role in the transcytotic pathway of Shh involving the Rab11-ARE compartment.

**FIGURE 8:**
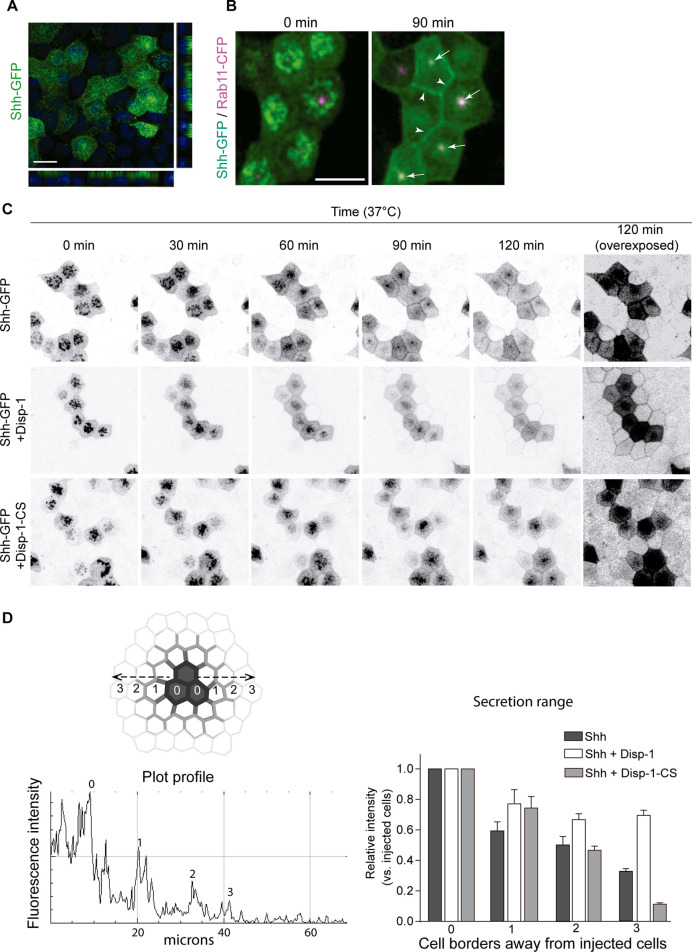
Live-cell imaging of Shh-GFP in cells coexpressing Disp-1 or Disp-1-CS. Polarized MDCK cells in glass coverslips were microinjected to express Shh-GFP alone or together with Disp-1 or Disp-1-CS. **(A)** Shh-GFP shows apical distribution after 4 h of expression. **(B)** Polarized MDCK cells stably expressing Rab11-CFP were microinjected with Shh-GFP plasmid and after 45 min of expression at 37°C, the cells were incubated for 2 h at 20°C to arrest trafficking of protein at the TGN (time = 0) and then shifted to 37°C to reestablish exit from the TGN for 90 min. Arrow heads show basolaterally arrived Shh-GFP, while arrows point to Rab11-ARE showing colocalization with Shh-GFP very likely reflecting its transcytotic route to the apical domain. **(C)** Polarized MDCK cells microinjected with indicated plasmids and treated as in B to accumulate Shh-GFP at the TGN were analyzed by live-cell imaging for the indicated times. Shh-GFP detected in the border of neighboring non-expressing cells reflects its basolateral secretion and diffusion, which was enhanced by co-expressed Disp-1 but not Disp-1-CS, as more clearly seen in the overexposed 120 min image. **(D)** Quantification of Shh fluorescence relative intensity at the cell borders of microinjected cells and their neighbors. A line was drawn from the center of an injected cell toward the surrounding non-injected cells. Plot-profile of the line measuring the fluorescence intensity shows peaks revealing cell borders, which are depicted in the graph as intensities relative to injected cells. Scale bar, 10 μm.

To further analyze the role of Disp-1 on biosynthetic trafficking of Shh, we performed live-cell imaging using Shh coupled to GFP (Shh-GFP) (Chamberlain et al., 2008). Plasmid-microinjected MDCK cells showed that Shh-GFP acquired an apical distribution and colocalization with Rab-11-ARE similar to the wild-type Shh (Figure 8B). Therefore, this construct gave the opportunity to follow the trafficking from the TGN to the cell surface in a synchronized manner using previously established methods (Kreitzer et al., 2003; Cancino et al., 2007; Guo et al., 2013). After plasmid microinjection, we incubated the cells for 45 min at 37°C to allow for detectable expression levels of Shh-GFP and then incubated the cells at 20°C for 2 h to arrest the biosynthetic trafficking at the TGN (Kreitzer et al., 2003; Cancino et al., 2007; Guo et al., 2013). At this initial time, Shh-GFP expressed alone or in combination with Disp-1 or Disp-1-CS mutant displayed the perinuclear pattern characteristic of the TGN (Figure 8C, 0 min). By shifting the cells to 37°C in the presence of cycloheximide to avoid further protein synthesis and follow post-TGN trafficking, we found that Shh-GFP became first detectable at the cell border and then progressively acquired the typical Rab11-ARE location and apical distribution (Figure 8C). A halo of receiving cells surrounding the expressing cells displayed fluorescent decoration in their borders revealing Shh-GFP basolateral secretion from producing cells (Figure 8C, upper panel). This can be seen more clearly in the overexposed image corresponding to the 120-min post-TGN trafficking (Figure 8C, right panel). The cells co-expressing Disp-1 showed enhanced Shh-GFP basolateral secretion (Figure 8C, middle panel, and Figure 8D), which according to the diffusing distance of Shh-GFP reached close to 40 microns (10 microns per cell) (Figure 8D). In contrast, coexpression of the Disp-1-CS mutant did not result in higher Shh-GFP basolateral secretion (Figure 8C, lower panel, and 8D). Disp-1-CS expressing cells showed more clearly accumulated Shh-GFP in the characteristic subapical punctate compartment (Figure 8C, lower panel). Taken together, all these experiments indicate that Disp-1 promotes Shh secretion first from the basolateral cell surface, where it arrives from the TGN, and is then required for trafficking along the basolateral-to-apical transcytosis route at the level of Rab11-ARE, where Shh accumulates if Disp-1 function is disturbed.

## 3 Discussion

In this study, we addressed the intracellular trafficking pathway and Disp-1-mediated secretion of newly synthesized Shh in polarized epithelial cells. We first show that stably transfected MDCK cells under steady-state conditions display and secrete Shh mainly apically. However, pulse-chase experiments show that newly synthesized Shh is first sorted to the basolateral cell surface and then reaches the apical cell surface. Cell surface tagging directly demonstrate Shh basolateral-to-apical transcytosis through a pathway involving the Rab11-ARE compartment. This assay also detects reverse transcytosis back to the basolateral cell surface. Determinants in this indirect route to the apical cell surface are the lipid adducts that attach Shh to the membrane. Live-cell imaging of plasmid-microinjected cells directly demonstrate transport of newly synthesized Shh from the TGN to the basolateral cell surface, showing also that Disp-1 promotes secretion at this stage of the Shh biosynthetic trafficking. The role of Disp-1 in the transcytotic pathway is then suggested by its detection together with Shh at Rab11-ARE, as well as by an increased colocalization of Shh in this compartment under coexpression of Disp-1-CS mutant. This complex intracellular trafficking offers multiples stages for regulation and predicts interdependence between apical and basolateral secretion in accordance with the Disp-1 expression levels. Variations in Shh polarized distribution and secretion described in different mammalian epithelial cells (Chamberlain et al., 2008; Therond, 2012; Mao et al., 2018) and debated models in *Drosophila* (Guerrero and Kornberg, 2014; Matusek et al., 2020) may be explained under this model of Shh sorting behavior.

Hhs have been found apically and/or basolaterally distributed and secreted with variable preponderance and signaling functions in diverse epithelia (Zavros, 2008; Briscoe and Therond, 2013; Guerrero and Kornberg, 2014; Peng et al., 2015; Shyer et al., 2015; Edeling et al., 2016; Petrov et al., 2017; Mao et al., 2018; Walton and Gumucio, 2021). An open question is whether Hh subcellular distribution and secretion reflects default-unsorted or specific-sorting trafficking events. MDCK cells possess apical and basolateral exocytic pathways roughly equivalent in transporting capacity, and therefore newly synthesized proteins require specific sorting signals to be addressed from the TGN to particular cell surface domains (Mellman and Nelson, 2008; Gonzalez and Rodriguez-Boulan, 2009; Rodriguez-Boulan and Macara, 2014). Proteins lacking sorting information follow these pathways by default resulting in unpolarized secretion in these cells (Gottlieb et al., 1986; Gonzalez et al., 1987; Scheiffele et al., 1995). In other epithelial cells, such as intestinal cells and hepatocytes, the exocytic route is prominently basolateral, and therefore most apical proteins are indirectly sorted by transcytosis from the basolateral cell surface, even though these cells also have a direct apical route (Bartles et al., 1987; Rindler and Traber, 1988; Hubbard, 1989; Hauri and Matter, 1991). Exogenous proteins that are unpolarized in MDCK cells can be basolaterally addressed in intestinal cells (Rindler and Traber, 1988). How the transporting capacity of apical and basolateral exocytic routes are organized in the most studied *Drosophila* epithelial cells is unknown (Guerrero and Kornberg, 2014; Matusek et al., 2020). Our findings in MDCK cells clearly indicate that Shh has sorting information for biosynthetic basolateral targeting. Therefore, it can be predicted that Shh would be initially sorted to the basolateral cell surface in most epithelial cells, including those having exocytic routes similar to MDCK or to intestinal cells.

Pulse-chase experiments show that after an initial basolateral sorting Shh gradually increases at the apical domain suggesting transcytosis. We confirmed this transcytosis by tagging the Shh at the basolateral cell surface with reducible biotin linkage (NHS-SS-Biotin) or with anti-Shh antibody. Following the fate of antibody-tagged Shh, we also show that its apical appearance is preceded by enrichment in a dense subapical structure, which according to its location and Rab11 staining very likely corresponds to the Rab11-ARE compartment (Apodaca et al., 1994; Barroso and Sztul, 1994; Casanova et al., 1999; Brown et al., 2000). We show that stably expressed Rab11-GFP redistributes from a perinuclear disperse location toward a subapical concentrated structure, while MDCK cells develop polarity. These different Rab11 distributions have been described in non-polarized and polarized cells (Casanova et al., 1999; Brown et al., 2000; Perez Bay et al., 2013; Perez Bay et al., 2016). In the basolateral-to-apical transcytosis pathway extensively characterized for pIgR, Rab11-ARE is located at the last stage, preceded by postendocytic trafficking along peripheral basolateral sorting endosomes (BSEs) and juxtanuclear common recycling endosomes (CREs) (Apodaca et al., 1994; Barroso and Sztul, 1994; Cardone et al., 1994; Wang et al., 2000a; Brown et al., 2000; Wang et al., 2000b; Golachowska et al., 2010). In polarized MDCK cells, Rab11-ARE is not only recognized by Rab11 staining but also by its characteristic dense morphology at subapical location and its enrichment in apically destined cargo (Brown et al., 2000; Perez Bay et al., 2013). A similar pathway follows the transferrin receptor in epithelial cells lacking the AP1B-dependent machinery that recycles basolateral proteins from CRE (Perez Bay et al., 2013). Transcytosis of NgCAM (neuron glia cell adhesion molecule) also involves Rab11-positive recycling endosomes (Thompson et al., 2007). It is interesting that distinct Rab GTPases have been shown to define subdomains in endosomal compartments, for example, Rab5, Rab4, and Rab11a in early endosomes (Sonnichsen et al., 2000) and Rab7 and Rab9 in late endosomes (Barbero et al., 2002). Thompson et al. (2007), using 3D imaging analysis of supranuclear recycling endosomes, described subdomains containing either apical or basolateral proteins, with Rab11 colocalizing with apical subdomains but more extensively with areas of overlap between apical and basolateral subdomains. Whether ARE and CRE are separate compartments or ARE belongs to a subdomain of CRE has been debated (Brown et al., 2000; Folsch et al., 2009; Golachowska et al., 2010). The finding of a different pH in ARE (pH = 6.5) and other recycling endosomes including “common endosomes” (pH = 5.8) favors the possibility of structurally separated compartments (Wang et al., 2000a). It is also worth to mention that the apical and basolateral subdomains, described by Thompson et al. (2007) developed along with polarization, are absent in non-polarized cells. We cannot ensure that fully endosomal subdomain maturation occurs in our coverslip-grown polarized cells. Even though we show Rab11 redistribution associated with the acquisition of a polarized phenotype, we do not know whether this process relates with the described subdomain maturation of recycling endosomes (Thompson et al., 2007). However, we show that Shh travelling to the apical domain involved Rab11-ARE in polarized MDCK cells grown in the filter, as well as in glass-coverslips, thus suggesting a similar functioning of at least this transcytotic route under both growing conditions.

For transmembrane proteins, basolateral-to-apical transcytosis reflects a differential use of sorting signals on endocytic and secretory pathways, as shown for pIgR (Luton et al., 2009), NgCAM (Anderson et al., 2005), and TfR (Perez Bay et al., 2014), remaining unknown for raft-associated luminal proteins such as Shh (Zurzolo and Simons, 2016; Parchure et al., 2018). On the other hand, our antibody-tagging assay also reveals that Shh undergoes reversed apical-to-basolateral transcytosis. This opposite transcytosis pathway has been mainly characterized for the FcRn receptor and includes ASE and CRE as sequential stations to the basolateral cell surface (Miettinen et al., 1992; Ramalingam et al., 2002; Golachowska et al., 2010; Jerdeva et al., 2010). Apical recycling proteins also use ASE and CRE but are instead directed to Rab11-ARE as the returning route to the apical cell surface (Brown et al., 2000; Perez Bay et al., 2016). Rab11-ARE has been described to receive also subsets of newly synthesized apical proteins by intersecting the biosynthetic pathway from the TGN (Weisz and Rodriguez-Boulan, 2009). The function of Rab11-ARE at all these crossroads probably adds regulation events by extracellular signaling to the last stage of apical sorting (Golachowska et al., 2010), as suggested for pIgR involving protein kinase C (Cardone and Mostov, 1995) and protein kinase A (Hansen and Casanova, 1994). ARE-like compartments are found in different epithelial cells (Goldenring et al., 1996). In gastric parietal cells, there is a precedent of an equivalent tubulovesicular structure containing both Rab11 (Calhoun et al., 1998) and Shh (Zavros et al., 2008), which is specialized in massive and externally controlled apical membrane recycling involving the actin cytoskeleton during acid secretion (Vial and Garrido, 1976; Gonzalez et al., 1981). Other endocytic compartments involved in transcytosis or recycling routes, such as BSE and CRE, possibly contribute to different forms of regulation, even at different stages within the same route, using distinct Rabs (e.g., Rab25 and Rab17) (Hunziker and Peters, 1998; Zacchi et al., 1998; Casanova et al., 1999; Wang et al., 2000b; Striz et al., 2018), microtubule motors (Schonteich et al., 2008; Perez Bay et al., 2013), and kinases (e.g., PI3K) (reviewed in Weisz and Rodriguez-Boulan (2009); Golachowska et al. (2010)). These possibilities deserve to be explored in Shh trafficking and function, both in health and disease.

Studies on *Drosophila* embryonic ectodermal cells suggest an initial Hh basolateral sorting followed by transcytosis to the apical domain (Gallet et al., 2003), congruent with the basolateral Hh accumulation found by silencing Disp (Gallet et al., 2003) or Rab8 (Gore et al., 2021). However, studies on the *Drosophila* wing disk epithelia propose that Hh is primarily sorted to the apical plasma membrane (Therond, 2012; Gradilla and Guerrero, 2013), being debated whether this is followed by apical recycling (D'Angelo et al., 2015) or apical-to-basolateral transcytosis (Callejo et al., 2011), accounting for long-range signaling. As we show in MDCK cells, predominant steady-state apical cell surface distribution and secretion of Shh can result from an indirect transcytosis route even when reverse transcytosis also occurs. On the other hand, apical recycling of Hh, described in the wing disk epithelia (D'Angelo et al., 2015), may in principle coexist with apical-to-basolateral transcytosis as proposed for megalin (McCarthy and Argraves, 2003; Perez Bay et al., 2016). Our proposed model of Shh trafficking is compatible with observations in *Drosophila* epithelial systems and perhaps may help to reinterpret and reconcile debated models (Guerrero and Kornberg, 2014; Matusek et al., 2020).

The trafficking behavior of Shh in MDCK cells implies that this morphogen has sorting information decoded first at the TGN and then at different stages of the transcytotic route. The finding that Shh mutant lacking palmitoylation and cholesteroylation is secreted unpolarized indicates that Shh peptide structure either lacks specific sorting signals for polarity or requires membrane association to expose sorting-competent proteinaceous signals. Another possibility is that lipids themselves direct the entire sorting process mediating association with particular membrane domains such as lipid rafts. We show that mutants lacking either palmitoylation (ShhNpC24S) or cholesteroylation (ShhN) predominantly distribute to the basolateral cell surface. Both lipid modifications seem to contribute to the basolateral sorting of Shh. We could not attribute a particular role of palmitoylation in the subsequent basolateral-to-apical trafficking because Shh defective in cholesterol modification is known to be inefficiently palmitoylated (Kohtz et al., 2001; Sbrogna et al., 2003; Chen et al., 2004; Feng et al., 2004). However, our pulse-chase and basolateral tagging experiments point to the cholesteryl group as a crucial determinant of Shh primary basolateral sorting and subsequent transcytosis.

Lipid-mediated association of Shh with lipid rafts (Long et al., 2015; Petrov et al., 2017; Parchure et al., 2018) is probably involved in its polarized sorting, though it is difficult to visualize a mechanism. Most GPI-APs are mainly addressed to the apical domain from the TGN through lipid raft association (Lisanti et al., 1988; Brown and Rose, 1992; Hua et al., 2006; Paladino et al., 2006; Imjeti et al., 2011; Zurzolo and Simons, 2016; Lebreton et al., 2019). Our pulse-chase experiments corroborate apical biosynthetic trafficking of a previously characterized recombinant GPI-AP (Imjeti et al., 2011), contrasting with Shh in the same type of an experiment. Lipid-raft association is certainly not enough for apical sorting (Paladino et al., 2004; Lebreton et al., 2019). An additional requirement found for GPI-APs is clustered at the TGN promoted by cholesterol and luminal calcium (Paladino et al., 2004; Lebreton et al., 2021). Also, glycosylation seems to be important in some epithelial cells (Imjeti et al., 2011). GPI-APs lacking such clustering properties are instead addressed to the basolateral domain, as shown for the prion protein (Sarnataro et al., 2002; Lebreton et al., 2021). The prion protein also becomes transcytosed to the apical domain (Arkhipenko et al., 2016). Lipid rafts exist in the basolateral cell surface at least associated with caveolins conforming to caveolae structures (Lahtinen et al., 2003) and are also presumably enriched in recycling endosomes (Gagescu et al., 2000), where the raft-agglomerating protein MAL2 has been involved in basolateral-to-apical transcytosis (de Marco et al., 2002). However, the mechanism through which the lipid-raft-associated proteins, such as prion and Shh, are basolaterally sorted, endocytosed, and addressed to the opposite domain by transcytosis remains unknown.

Finally, we explored the distribution and Shh secretion function of Disp-1 using an approach that evaluates recently synthesized proteins. Stewart et al. (2018) described that Disp-1 acquires functional competence after furin-mediated cleavage, proposed to occur at the cell surface. In MDCK cells, transient transfection experiments show Disp-1 distributed to the basolateral region due to sorting information contained in its intracellular region (Etheridge et al., 2010). Even though we found the expression of Disp-1 in MDCK cells, the lack of suitable antibodies and the intolerance of these cells to prolonged Disp-1 overexpression prompted us to assess its location and secretion function through plasmid microinjection experiments. We show that recently synthesized Disp-1 predominantly distributes to the subapical region, just underneath the cell surface located Shh. Slight levels of both Disp-1 and Shh can also be detected at the basolateral border. In *Drosophila*, exogenously expressed Disp has been reported in some studies to be enriched in the plasma membrane of apical, basal, and lateral regions (D'Angelo et al., 2015), contrasting with other studies that locate this protein mainly at the basolateral plasma membrane (Callejo et al., 2011; Stewart et al., 2018). All these observations most likely reflect a dynamic Disp-1 trafficking and variations depending on its expression levels and experimental conditions. Our plasmid microinjection experiments combined with live-cell imaging show that an increased expression of Disp-1 enhances basolateral secretion of newly synthesized Shh coming from the TGN. Etheridge et al. (2010) described a decreased basolateral secretion of Shh under conditions of Disp-1 silencing or Disp-1 mutant expression in MDCK cells. We find that coexpressed Disp-CS mutant (Stewart et al., 2018) does not increase the basolateral secretion of Shh after exiting the TGN. Indeed, Disp-1 is also expected to mediate apical Shh secretion, as it does upon Hh in *Drosophila* (D'Angelo et al., 2015). However, this is difficult to assess in our microinjection experiments and was neither evaluated in previous studies (Etheridge et al., 2010). A trade-off between apical and basolateral Hh secretion has been proposed in the wing disk epithelia (Ayers et al., 2010; Gore et al., 2021). Our results would support this possibility considering that Disp-1 exerts its secretion function first at the basolateral cell surface and Shh then undergoes transcytosis to the apical domain.

Disp has also been proposed to play a role in Hhs subcellular distribution and trafficking somehow acting on endocytic pathways (Stewart et al., 2018; Pizette et al., 2021; D'Angelo et al., 2015; Callejo et al., 2011). *Drosophila* model systems have shown that Hh secretion and signaling are sensitive to alterations in several of the small GTPases that regulate endosomal trafficking, such as Rab4, Rab5, Rab8, and Rab11 (D'Angelo et al., 2015; Callejo et al., 2011; Gore et al., 2021; Pizette et al., 2021; Stewart et al., 2018). The effects of Disp-1-CS expression suggest a requirement of Disp-1 cleavage in endosomal membrane trafficking through yet unknown mechanisms (Stewart et al., 2018). Recent evidence in *Drosophila* highlights a role of Disp and Rab11 in Hh delivery from a tubular endosomal compartment that was named Hherisome (Pizette et al., 2021). Whether Hherisome and Rab11-ARE are equivalent compartments remains unknown. Our microinjection experiments revealed colocalization of newly synthesized Disp-1 and Shh at Rab11-ARE. Furthermore, the colocalization of Rab11 with Shh increased under Disp-1-CS coexpression suggesting a delayed traffic through Rab11-ARE. These results suggest that Disp-1 function is required for Shh trafficking along the basolateral-to-apical transcytosis pathway of recently synthesized Shh at the level of the Rab11-ARE compartment.

Hhs have been found secreted in different ways, including multimers (Zeng et al., 2001; Gallet et al., 2006), lipoprotein particles (Panakova et al., 2005), extracellular vesicles (Tanaka et al., 2005; Gradilla et al., 2014; Matusek et al., 2014; Vyas et al., 2014; Parchure et al., 2015), and associated with long filopodia called cytonemes (Roy et al., 2011; Hall et al., 2021; Gradilla and Guerrero, 2022). Hh proteins have been also shown to form nano-scaled oligomers *via* electrostatic interactions, and this process is required for endocytosis followed by targeting multivesicular bodies and secretion in exosomes (Vyas et al., 2008; Vyas et al., 2014; Parchure et al., 2015). How our proposed model of Shh basolateral sorting followed by transcytosis relates with particular Shh forms of secretion and oligomerization poses interesting challenges for future studies, especially regarding potential links with morphogenesis and cancer.

## 4 Materials and methods

### 4.1 Cloning and subcloning

cDNA plasmid coding for Shh (pBlueScript-Shh), ShhNpC24S (pSK-C24S), and ShhNC24S (pRK5-ShhNC24S), kindly provided by Philip A. Beachy, Stanford University, CA, United States, were subcloned by double digestion with EcoRI and XbaI (for Shh), NotI (for ShhNpC24S), or NotI and KpnI (for ShhNC24S) restriction enzymes and ligated into pcDNA3.1(-) with T4 DNA ligase (Thermo Fisher Cat #15224017) according to the manufacturer’s instructions. ShhN dsDNA coding for residues 1–198 of murine Shh was obtained by PCR using Pfu polymerase (Promega Cat #M7741), and the following primers were used: Fw: GTC​TCG​AGC​CAA​CTC​CGA​TGT​GTT​CCG and Rev: CGG​AAT​TCT​TAG​CCG​CCG​GAT​TTG​GCC that include XhoI and EcoRI sites for subcloning into pcDNA3.1(+). All constructs were confirmed by sequencing.

### 4.2 Cell lines and transfection

Type II MDCK cells were grown in DMEM (Cat# 12100046, Thermo Fisher) supplemented with 7.5% fetal bovine serum (FBS, Cat# SH30071.03 HyClone), 50 U/mL antibiotics (penicillin–streptomycin mix (Cat# 15070063, Thermo Fisher), and 5 μg/ml Plasmocin (Cat# ant-mpp, InvivoGen) in a 37°C, 5% CO_2_ humidified incubator, routinely analyzed by RT-PCR for *Mycoplasma* contamination. Passages were made before reaching confluence every 2–3 days by two washes with PBS (15 min each) and incubation in 0.05% trypsin for 5–10 min (trypsin Cat# 15400054, Thermo Fisher). Stable transfected clones MDCK-Shh, -ShhN, -ShhNC24S, and -ShhNpC24S were generated by transfecting 100.000 cells with 1 μg of plasmid DNA and Lipofectamine 2000 reagent in 24-multiwell plates. The cells were trypsinized at the next day and plated at 1/10 and 1/100 dilutions into 100-mm dishes for selection with G418 (0.8 mg/ml), which was changed every 5 days until visible colonies were isolated using cloning cylinders and expanded in six-multiwell plates. Screening was made by immunofluorescence (see below) and immunoblot of lysates and media.

### 4.3 Protein analysis

Cell lysis: The cells were washed twice in PBS containing 1 mM MgCl_2_ and 0.1 mM CaCl_2_ (PBS-CM) and lysed in 500 µL RIPA buffer (50 mM Tris–HCl (pH 8.0), 150 mM NaCl, 0.5% Na-deoxycholate, 0.1% SDS, and 1% Triton X-100) supplemented with protease inhibitors (PMSF 2 mM, leupeptin 2 μg/ml, and pepstatin 1 μg/ml). The cell lysates were cleared by centrifugation at 14.000 rpm at 4°C, and the protein concentration was determined using the BCA method. Immunoprecipitation: The key resources table indicates the amount of antibodies (GFP, E-cadherin, and 5E1) used per sample. Antibodies were pre-incubated with 30 µL of protein-A–agarose for 2 h at 4°C, and then beads were washed three times to remove excess antibodies. Total cell lysate (approx. 500 µg) or collected medium was then added to beads and incubated for 2 h at 4°C, washed three times in RIPA buffer, and eluted in 30 µL of 2X Laemmli Buffer. Immunoblot: Equal protein amounts were separated by SDS-PAGE (12%) followed by wet-transfer onto PVDF membranes.

### 4.4 Steady-state Shh secretion

Proteins of 2–6 h conditioned in low-serum media (0.3% FBS) were precipitated by 10% trichloroacetic acid (TCA) for 30 min on ice, centrifuged at 4°C and 14.000 rpm for 10 min, washed once with 1 ml of 100% acetone, and re-centrifuged. Pellets were solubilized in 30 μL of NaOH 0.1M and 10 μL of 3X Laemmli sample buffer, resolved in SDS-PAGE (12%), transferred to PVDF membranes, and immunoblotted with the indicated antibodies.

### 4.5 Polarized MDCK cells

MDCK cells polarized in Transwell inserts: MDCK cells (100,000–400,000 cells) were plated on 12-mm or 24-mm polycarbonate Transwell inserts with 0.4 mm pore size (Costar) and grown with daily media changes until transepithelial resistance reached 400 ohm x cm^2^ (4–5 days), as measured using an EVOM electrometer (World Precision Instruments, Sarasota, FL). MDCK cells polarized in glass coverslips: MDCK cells (5000 cells) were plated in 12-mm-diameter glass coverslips and used for the experiments 3–4 days after achieving full confluence. Under both conditions, the cells developed primary cilia, Rab11-ARE punctate compartment subapically localized, and distribution of basolateral markers, as characteristics of the polarized phenotype (Casanova et al., 1999; Brown et al., 2000; Folsch et al., 2009; Perez Bay et al., 2013).

### 4.6 Immunofluorescence

Cells were fixed in freshly prepared 4% PFA in PBS-CM for 15 min at room temperature or overnight at 4°C, and permeabilization was performed with 0.2% saponin in PBS-CM as indicated in the corresponding experiment. All subsequent steps contained 0.2% saponin. Primary and secondary antibodies were incubated for 30 min at 37°C or overnight at 4°C. Coverslips were mounted in Fluoromount.

### 4.7 Domain-selective surface biotinylation

Steady-state apical distribution and basolateral distribution of Shh and endogenous proteins were analyzed by domain-selective biotinylation of MDCK cells grown in Transwell inserts, as described in Bravo-Zehnder et al. (2000) and Oyanadel et al. (2018). The cells were rinsed twice with ice-cold PBS-CM followed by two successive 20 min incubations at 4°C with 0.5 mg/ml Sulfo-NHS-LC-Biotin in PBS-CM, added to the apical (0.6 ml) or basolateral (1 ml) sides. The cells were then incubated with 50 mM NH_4_Cl in PBS-CM for 10 min, rinsed twice with PBS-CM, and lysed in RIPA buffer, supplemented with protease inhibitors for 30 min at 4°C. NeutrAvidin–Agarose was used to retrieve biotinylated proteins by centrifugation (18,000 x g for 10 min), which then were analyzed by immunoblot against Shh and endogenous proteins, as described in Sargiacomo et al. (1989), Bravo-Zehnder et al. (2000) and Oyanadel et al. (2018).

### 4.8 Pulse-chase domain-selective targeting assay of newly synthesized proteins

Stable transfected cells were pulse-labeled in methionine-free and cysteine-free DMEM supplemented with 400 μCi/ml [^35^S]-methionine/cysteine for 30 min in 120 μL of the medium applied to the basolateral side of the inverted filter, as described in Marzolo et al. (1997). Chase for different times was made in the growth media supplemented with 3- to 6-fold excess cold methionine (0.6 mM) and cysteine and (1.2 mM) in the presence of 350 μM cycloheximide. The cells were chilled, domain-selective surface biotinylated in ice-cold PBS-CM, and lysed with RIPA buffer supplemented with protease inhibitors at 4°C for 30 min. Lysates were cleared by centrifugation (18,000 x g for 10 min) before immunoprecipitation with protein-A-agarose-preincubated antibodies (5E1-anti-Shh or E-cadherin). The mixture of biotinylated (surface) plus non-biotinylated (intracellular) immunoprecipitated proteins was incubated at 95°C for 5 min in 30 μL of 10% SDS RIPA to dissociate immune complexes. The eluted proteins were diluted to 1 ml with RIPA and subjected to NeutrAvidin–Agarose precipitation to isolate surface-located Shh. Secreted Shh was immunoprecipitated from the apical and basolateral media with protein-A–Sepharose-bound 5E1, as described for other proteins (Gonzalez et al., 1987; Gonzalez et al., 1993; Marzolo et al., 1997). The immune complexes of surface-located and secreted Shh were resolved by SDS-PAGE, and [^35^S]-methionine/cysteine-labeled proteins were developed in the Cyclone Plus scanner (PerkinElmer) using OptiQuant software (3.0, PerkinElmer).

### 4.9 Transcytosis assays

Trafficking of Shh from one cell surface domain to the opposite (transcytosis) was analyzed by domain-specific biotinylation and cell surface-tagging immunofluorescence assays.

#### 4.9.1 Biotinylation transcytosis assay

Biotinylation transcytosis assay was performed with the reducible biotin reagent Sulfo-NHS-SS-Biotin, as described in Burgos et al. (2004). In brief, six Transwell filter cups with polarized live MDCK monolayer cells were subjected to basolateral-specific biotinylation with Sulfo-NHS-SS-Biotin in PBS-CM*.* One filter cup was used for total basolateral biotinylated Shh (“non-reduced, time 0”), and other two filter cups were subjected to either apical or basolateral reduction with 22 mM MesNa reagent (0 min) made in 50 mM Tris–HCl buffer, pH 8.6 and 100 mM NaCl. The remaining three filter cups were incubated for 120 min at 37°C in growth media to allow trafficking, and then MesNa was added to the apical or basolateral side. Monolayers were alkylated with 20 mM iodoacetamide in PBS-CM containing 1% BSA, washed twice in PBS-CM, lysed in RIPA buffer supplemented with protease inhibitors, and subjected to NeutrAvidin–Sepharose precipitation and immunoblot. To analyze apical secretion of Shh from the basolateral membrane, the cells were biotinylated at the basolateral side and then incubated in DMEM containing 0.5% fetal bovine serum at 37°C for 2 h. Biotinylated Shh proteins were retrieved from apical and basolateral media with NeutrAvidin–Agarose and immunoblotted.

#### 4.9.2 Cell surface-tagging immunofluorescence transcytosis assay

Live MDCK cells grown in Transwell inserts were apically or basolaterally incubated with 5E1 antibody diluted in DMEM (supplemented with 10 mM HEPES) for 30 min at 4°C. After three washes in PBS-CM, the cells were shifted to 37°C in growth media to allow trafficking for different times, fixed with PFA-PBS-CM, and permeabilized. Shh bound to the primary antibody was detected with fluorophore-coupled secondary antibodies for 30 min at 37°C.

#### 4.9.3 Secretion of transcytosed ShhNpC24S

MDCK cells grown in Transwell inserts were rinsed twice with ice-cold PBS-CM followed by two successive 20 min incubations at 4°C with 0.5 mg/ml Sulfo-NHS-LC-Biotin in PBS-CM, added to the basolateral side. The cells were then incubated with 50 mM NH_4_Cl in PBS-CM for 5 min, rinsed twice with PBS-CM, and shifted to 37°C in growth media for 2 h. The apical and basolateral media were collected, precipitated with NeutrAvidin–Agarose, and analyzed by immunoblot.

### 4.10 Microinjection

Polarized MDCK cells grown in filters or glass coverslips were intranuclearly microinjected with expression plasmids using back-loaded glass capillaries in the Eppendorf NI2 micromanipulator coupled to the Eppendorf Transjector 5246 system. Expression plasmid cDNAs were used at a concentration of 20–100 ng/μL in 10 mM HEPES buffer, pH 7.4 and 140 mM KCl. The cells were maintained in DMEM-HEPES during microinjection.

### 4.11 Live-cell imaging

The microinjection approach to express and accumulate exogenous proteins at the TGN and then assess subsequent trafficking was performed as described in Kreitzer et al. (2000) and Cancino et al. (2007). Briefly, microinjected cells were incubated for 45 min at 37°C in DMEM-HEPES plus 7.5% FBS to allow protein expression. This time was selected to minimize endoplasmic reticulum leakage. The plasmid concentration was adjusted (20–100 ng/μL) for fluorescence microscopy-detectable protein expression. The exocytic trafficking was then arrested at the TGN by incubating the cells at 20°C for 2 h in DMEM-HEPES in the presence of cycloheximide (350 μM). After the 20°C block, the cells were mounted on a Leica SP8 microscope in DMEM-HEPES plus cycloheximide and incubated at 37°C to resume trafficking.

### 4.12 Images acquisition and processing

Images were collected at 1024 × 1024 pixel resolution in Z-stacks of 300-nm steps using a Leica SP8 confocal microscope and a 63× oil immersion 1.4 ma lens and processed using ImageJ software, NIH, available for free. High-resolution images were acquired at Nyquist rate parameters (Nyquist Calculator, SVI, NL), and the resulting oversampled images were deconvolved using Huygens Essential software (SVI, NL), which was also used for 3D surface rendering processing.

### 4.13 Statistical analysis

Significance was determined using Prism software, and Student’s t-test was used to compare the mean (*p ≤ 0.05; **p ≤ 0.01; ***p ≤ 0.001). Error bars indicate the standard error of mean (SEM).

### 4.14 Key resources table

Reagents and their sources are shown in Table 1.

**TABLE 1:** Key resources table.

Reagent type (species) or resource	Designation	Source or reference	Identifier	Additional information
Cell line (*Canis familiaris*)	MDCK (Madin–Darby canine kidney) II cells	ATCC provided by Enrique Rodriguez-Boulan (Weill Cornell Medical College, New York, United States)		
Culture media	DMEM	Thermo Fisher	12100046	
Culture media	Methionine- and cysteine-free DMEM	Thermo Fisher	21013024	For metabolic label
Culture media	DEMEM-HEPES	Thermo Fisher	12430054	For microinjection and live-cell imaging
Transfected/microinjected construct (*M. musculus*)	pCDNA3.1-Shh	This paper		Subcloned from pBluescript-mShh (P. A. Beachy’s laboratory) (Chang et al., 1994)
Transfected construct (*M. musculus*)	pCDNA3.1-ShhNC24S	This article		Subcloned from pRK5-mShhNC24S (P. A. Beachy’s laboratory) (Maity et al., 2005)
Transfected construct (*M. musculus*)	pCDNA3.1-ShhN	This article		PCR cloning from pCDNA3.1-Shh
Transfected construct (*M. musculus*)	pCDNA3.1-ShhNpC24S	This article		Subcloned from pSK-C24S (P. A. Beachy’s laboratory) Williams et al. (1999)
Microinjected construct (*M. musculus*)	pCDNA3-Shh-GFP	McMahon’s Laboratory (Chamberlain et al., 2008)		
Cell line (Canis familiaris)	MDCK-Rab11-CFP	This article		Stably expressing human Rab11a-mCherry
Cell line (Canis familiaris)	MDCK-GFP-NO-GPI	A. Gonzalez’s Laboratory (Imjeti et al., 2011)		Stably expressing GFP-NO-GPI
Microinjected construct (*M. musculus*)	pCDNA3 V5-Dispatched-HA	S. Ogden’s Laboratory (Stewart et al., 2018)		pCDNA3 from Invitrogen
Microinjected construct (*M. musculus*)	pCDNA3 V5-Dispatched-CS-HA	S. Ogden’s Laboratory (Stewart et al., 2018)		pCDNA3 from Invitrogen
Microinjected construct (*M. musculus*)	LDLR-GFP-Y18A	K. Matter’s Laboratory (Hunziker et al., 1991)		pCB6-LDLR-GFP-Y18A
Antibody	Anti-Shh	DSHB (Developmental Studies Hybridoma Bank)	5E1	IF (1:50)
				IP (1:50)
Antibody	Anti-Shh	Santa Cruz Biotechnology, Inc. (California, United States)	sc-9024 (H-160)	WB (1:1000)
Antibody	Anti-E-cadherin	Cell Signaling	24E10 mAb #3195	IF (1:100)
Antibody	Anti-E-cadherin	BD Transduction Laboratories		IP (2mg/sample)
Antibody	Anti-GFP (rabbit)	This article		IP (1:250)
Antibody	Anti-Rab11a (rabbit)	Thermo Fisher Scientific	715,300	IF (1:100)
Antibody	Anti-fibronectin	Sigma	F6140	WB (1:2000)
Antibody	Anti-Na^+^/K^+^-ATPase	Santa Cruz Biotechnology, Inc. (California, United States)	sc-21713	WB (1:2000)
Antibody	Anti-HA (chicken)	Millipore	AB3254	IF (1:100)
Antibody	AlexaFluor 488	Life Technologies, Carlsbad, CA	A11029 (mouse)	1:500
			A11034 (rabbit)	
			A-11039 (chicken)	
Antibody	AlexaFluor 555	Life Technologies	A21424 (mouse); A21429 (rabbit)	1:500
			A32932 (chicken)	
Antibody	AlexaFluor 647	Life Technologies	A21235 (mouse)	1:500
Fluorescent dye	Hoechst	Thermo Fisher Scientific	62,249	1:1000
Software, algorithm	Adobe Photoshop CS4	Adobe, San Jose, CA		For making figures
Software, algorithm	LAS X	Leica, Wetzlar, Germany		Image analysis
Software, algorithm	Prism	GraphPad, La Jolla, CA		For statistical, analysis, and graphs
Software, algorithm	SigmaPlot10 software			For quantification of Golgi retention
Software, algorithm	Huygens Professional software			For deconvolution images
Software, algorithm	ImageJ (NIH)		2.0.0-rc-69/1.52o	Image analysis
Software, algorithm	OptiQuant software	PerkinElmer	3.0	
Commercial assay or kit	Lipofectamine 2000	Thermo Fisher Scientific	11668027	
Commercial assay or kit	Pierce™ ECL Western Blotting Substrate	Pierce^TM^, Waltham, MA, United States	32106	
Chemical compound, drug	Sulfo-NHS-LC-biotin	Pierce^TM^ /Thermo Fisher Scientific	21335	
Chemical compound, drug	EZ-Link™ NHS-SS-Biotin	Pierce/Thermo Fisher Scientific	21441	
Chemical compound, drug	NeutrAvidin–Agarose	Pierce/Thermo Fisher Scientific	29201	
Chemical compound, drug	MesNa	Sigma	PHR1570	
Chemical compound, drug	^35^S-methionine/cysteine	PerkinElmer NEN Life Science Products (Boston, United States)	NEG709A005MC	
Chemical compound, drug	Fluoromount-G	Thermo Fisher Scientific	00495802	
Polarized culture chamber	24 mm Transwells	Costar	3412	For biochemistry
Polarized culture chamber	12 mm Transwells	Costar	3401	For immunofluorescence
Polarized culture chamber	12 mm Snapwells	Costar	3801	For microinjection

## Data Availability

The original contributions presented in the study are included in the article/Supplementary Material; further inquiries can be directed to the corresponding author.

## References

[B1] Anderson E. Maday S. Sfakianos J. Hull M. Winckler B. Sheff D. (2005). Transcytosis of NgCAM in epithelial cells reflects differential signal recognition on the endocytic and secretory pathways. J. Cell Biol. 170, 595–605. 10.1083/jcb.200506051 16087710 PMC2171499

[B2] Ang A. L. Taguchi T. Francis S. Folsch H. Murrells L. J. Pypaert M. (2004). Recycling endosomes can serve as intermediates during transport from the Golgi to the plasma membrane of MDCK cells. J. Cell Biol. 167, 531–543. 10.1083/jcb.200408165 15534004 PMC2172492

[B3] Apodaca G. Katz L. A. Mostov K. E. (1994). Receptor-mediated transcytosis of IgA in MDCK cells is via apical recycling endosomes. J. Cell Biol. 125, 67–86. 10.1083/jcb.125.1.67 8138576 PMC2120019

[B4] Arkhipenko A. Syan S. Victoria G. S. Lebreton S. Zurzolo C. (2016). PrPC undergoes basal to apical transcytosis in polarized epithelial MDCK cells. PLoS One 11, e0157991. 10.1371/journal.pone.0157991 27389581 PMC4936696

[B5] Ayers K. L. Gallet A. Staccini-Lavenant L. Therond P. P. (2010). The long-range activity of Hedgehog is regulated in the apical extracellular space by the glypican Dally and the hydrolase Notum. Dev. Cell 18, 605–620. 10.1016/j.devcel.2010.02.015 20412775

[B6] Barbero P. Bittova L. Pfeffer S. R. (2002). Visualization of Rab9-mediated vesicle transport from endosomes to the trans-Golgi in living cells. J. Cell Biol. 156, 511–518. 10.1083/jcb.200109030 11827983 PMC2173336

[B7] Barroso M. Sztul E. S. (1994). Basolateral to apical transcytosis in polarized cells is indirect and involves BFA and trimeric G protein sensitive passage through the apical endosome. J. Cell Biol. 124, 83–100. 10.1083/jcb.124.1.83 7905002 PMC2119901

[B8] Bartles J. R. Feracci H. M. Stieger B. Hubbard A. L. (1987). Biogenesis of the rat hepatocyte plasma membrane *in vivo*: Comparison of the pathways taken by apical and basolateral proteins using subcellular fractionation. J. Cell Biol. 105, 1241–1251. 10.1083/jcb.105.3.1241 3654750 PMC2114787

[B9] Beachy P. A. Karhadkar S. S. Berman D. M. (2004). Tissue repair and stem cell renewal in carcinogenesis. Nature 432, 324–331. 10.1038/nature03100 15549094

[B10] Bravo-Zehnder M. Orio P. Norambuena A. Wallner M. Meera P. Toro L. (2000). Apical sorting of a voltage- and Ca2+-activated K+ channel alpha -subunit in Madin-Darby canine kidney cells is independent of N-glycosylation. Proc. Natl. Acad. Sci. U. S. A. 97, 13114–13119. 10.1073/pnas.240455697 11069304 PMC27187

[B11] Briscoe J. Therond P. P. (2013). The mechanisms of Hedgehog signalling and its roles in development and disease. Nat. Rev. Mol. Cell Biol. 14, 416–429. 10.1038/nrm3598 23719536

[B12] Brown D. A. Rose J. K. (1992). Sorting of GPI-anchored proteins to glycolipid-enriched membrane subdomains during transport to the apical cell surface. Cell 68, 533–544. 10.1016/0092-8674(92)90189-j 1531449

[B13] Brown P. S. Wang E. Aroeti B. Chapin S. J. Mostov K. E. Dunn K. W. (2000). Definition of distinct compartments in polarized Madin-Darby canine kidney (MDCK) cells for membrane-volume sorting, polarized sorting and apical recycling. Traffic 1, 124–140. 10.1034/j.1600-0854.2000.010205.x 11208093

[B14] Burgos P. V. Klattenhoff C. De La Fuente E. Rigotti A. Gonzalez A. (2004). Cholesterol depletion induces PKA-mediated basolateral-to-apical transcytosis of the scavenger receptor class B type I in MDCK cells. Proc. Natl. Acad. Sci. U. S. A. 101, 3845–3850. 10.1073/pnas.0400295101 15007173 PMC374332

[B15] Burke R. Nellen D. Bellotto M. Hafen E. Senti K. A. Dickson B. J. (1999). Dispatched, a novel sterol-sensing domain protein dedicated to the release of cholesterol-modified hedgehog from signaling cells. Cell 99, 803–815. 10.1016/s0092-8674(00)81677-3 10619433

[B16] Calhoun B. C. Lapierre L. A. Chew C. S. Goldenring J. R. (1998). Rab11a redistributes to apical secretory canaliculus during stimulation of gastric parietal cells. Am. J. Physiol. 275, C163–C170. 10.1152/ajpcell.1998.275.1.C163 9688847

[B17] Callejo A. Bilioni A. Mollica E. Gorfinkiel N. Andres G. Ibanez C. (2011). Dispatched mediates Hedgehog basolateral release to form the long-range morphogenetic gradient in the Drosophila wing disk epithelium. Proc. Natl. Acad. Sci. U. S. A. 108, 12591–12598. 10.1073/pnas.1106881108 21690386 PMC3150953

[B18] Cancino J. Torrealba C. Soza A. Yuseff M. I. Gravotta D. Henklein P. (2007). Antibody to AP1B adaptor blocks biosynthetic and recycling routes of basolateral proteins at recycling endosomes. Mol. Biol. Cell 18, 4872–4884. 10.1091/mbc.e07-06-0563 17881725 PMC2096610

[B19] Cannac F. Qi C. Falschlunger J. Hausmann G. Basler K. Korkhov V. M. (2020). Cryo-EM structure of the Hedgehog release protein Dispatched. Sci. Adv. 6, eaay7928. 10.1126/sciadv.aay7928 32494603 PMC7159904

[B20] Cardone M. H. Smith B. L. Song W. Mochly-Rosen D. Mostov K. E. (1994). Phorbol myristate acetate-mediated stimulation of transcytosis and apical recycling in MDCK cells. J. Cell Biol. 124, 717–727. 10.1083/jcb.124.5.717 8120094 PMC2119954

[B21] Cardone M. Mostov K. (1995). Wortmannin inhibits transcytosis of dimeric IgA by the polymeric immunoglobulin receptor. FEBS Lett. 376, 74–76. 10.1016/0014-5793(95)01251-8 8521971

[B22] Casanova J. E. Wang X. Kumar R. Bhartur S. G. Navarre J. Woodrum J. E. (1999). Association of Rab25 and Rab11a with the apical recycling system of polarized Madin-Darby canine kidney cells. Mol. Biol. Cell 10, 47–61. 10.1091/mbc.10.1.47 9880326 PMC25153

[B23] Caspary T. Garcia-Garcia M. J. Huangfu D. Eggenschwiler J. T. Wyler M. R. Rakeman A. S. (2002). Mouse Dispatched homolog1 is required for long-range, but not juxtacrine, Hh signaling. Curr. Biol. 12, 1628–1632. 10.1016/s0960-9822(02)01147-8 12372258

[B24] Chamberlain C. E. Jeong J. Guo C. Allen B. L. Mcmahon A. P. (2008). Notochord-derived Shh concentrates in close association with the apically positioned basal body in neural target cells and forms a dynamic gradient during neural patterning. Development 135, 1097–1106. 10.1242/dev.013086 18272593

[B25] Chang D. T. Lopez A. Von Kessler D. P. Chiang C. Simandl B. K. Zhao R. (1994). Products, genetic linkage and limb patterning activity of a murine hedgehog gene. Development 120, 3339–3353. 10.1242/dev.120.11.3339 7720571

[B26] Chen H. Liu Y. Li X. (2020). Structure of human Dispatched-1 provides insights into Hedgehog ligand biogenesis. Life Sci. Alliance 3, e202000776. 10.26508/lsa.202000776 32646883 PMC7362390

[B27] Chen M. H. Li Y. J. Kawakami T. Xu S. M. Chuang P. T. (2004). Palmitoylation is required for the production of a soluble multimeric Hedgehog protein complex and long-range signaling in vertebrates. Genes Dev. 18, 641–659. 10.1101/gad.1185804 15075292 PMC387240

[B28] Chen X. Tukachinsky H. Huang C. H. Jao C. Chu Y. R. Tang H. Y. (2011). Processing and turnover of the Hedgehog protein in the endoplasmic reticulum. J. Cell Biol. 192, 825–838. 10.1083/jcb.201008090 21357747 PMC3051819

[B29] Creanga A. Glenn T. D. Mann R. K. Saunders A. M. Talbot W. S. Beachy P. A. (2012). Scube/You activity mediates release of dually lipid-modified Hedgehog signal in soluble form. Genes Dev. 26, 1312–1325. 10.1101/gad.191866.112 22677548 PMC3387659

[B30] Cresawn K. O. Potter B. A. Oztan A. Guerriero C. J. Ihrke G. Goldenring J. R. (2007). Differential involvement of endocytic compartments in the biosynthetic traffic of apical proteins. EMBO J. 26, 3737–3748. 10.1038/sj.emboj.7601813 17673908 PMC1952228

[B31] D'Angelo G. Matusek T. Pizette S. Therond P. P. (2015). Endocytosis of Hedgehog through dispatched regulates long-range signaling. Dev. Cell 32, 290–303. 10.1016/j.devcel.2014.12.004 25619925

[B32] De Marco M. C. Martin-Belmonte F. Kremer L. Albar J. P. Correas I. Vaerman J. P. (2002). MAL2, a novel raft protein of the MAL family, is an essential component of the machinery for transcytosis in hepatoma HepG2 cells. J. Cell Biol. 159, 37–44. 10.1083/jcb.200206033 12370246 PMC2173496

[B33] Deborde S. Perret E. Gravotta D. Deora A. Salvarezza S. Schreiner R. (2008). Clathrin is a key regulator of basolateral polarity. Nature 452, 719–723. 10.1038/nature06828 18401403 PMC4078870

[B34] Donoso M. Cancino J. Lee J. Van Kerkhof P. Retamal C. Bu G. (2009). Polarized traffic of LRP1 involves AP1B and SNX17 operating on Y-dependent sorting motifs in different pathways. Mol. Biol. Cell 20, 481–497. 10.1091/mbc.e08-08-0805 19005208 PMC2613102

[B35] Edeling M. Ragi G. Huang S. Pavenstadt H. Susztak K. (2016). Developmental signalling pathways in renal fibrosis: The roles of notch, wnt and hedgehog. Nat. Rev. Nephrol. 12, 426–439. 10.1038/nrneph.2016.54 27140856 PMC5529143

[B36] Etheridge L. A. Crawford T. Q. Zhang S. Roelink H. (2010). Evidence for a role of vertebrate Disp1 in long-range Shh signaling. Development 137, 133–140. 10.1242/dev.043547 20023168 PMC2796928

[B37] Feng J. White B. Tyurina O. V. Guner B. Larson T. Lee H. Y. (2004). Synergistic and antagonistic roles of the Sonic hedgehog N- and C-terminal lipids. Development 131, 4357–4370. 10.1242/dev.01301 15294867

[B38] Folsch H. Mattila P. E. Weisz O. A. (2009). Taking the scenic route: Biosynthetic traffic to the plasma membrane in polarized epithelial cells. Traffic 10, 972–981. 10.1111/j.1600-0854.2009.00927.x 19453969 PMC2786770

[B39] Folsch H. Ohno H. Bonifacino J. S. Mellman I. (1999). A novel clathrin adaptor complex mediates basolateral targeting in polarized epithelial cells. Cell 99, 189–198. 10.1016/s0092-8674(00)81650-5 10535737

[B40] Gagescu R. Demaurex N. Parton R. G. Hunziker W. Huber L. A. Gruenberg J. (2000). The recycling endosome of Madin-Darby canine kidney cells is a mildly acidic compartment rich in raft components. Mol. Biol. Cell 11, 2775–2791. 10.1091/mbc.11.8.2775 10930469 PMC14955

[B41] Gallet A. Rodriguez R. Ruel L. Therond P. P. (2003). Cholesterol modification of hedgehog is required for trafficking and movement, revealing an asymmetric cellular response to hedgehog. Dev. Cell 4, 191–204. 10.1016/s1534-5807(03)00031-5 12586063

[B42] Gallet A. Ruel L. Staccini-Lavenant L. Therond P. P. (2006). Cholesterol modification is necessary for controlled planar long-range activity of Hedgehog in Drosophila epithelia. Development 133, 407–418. 10.1242/dev.02212 16396912

[B43] Gan Y. Mcgraw T. E. Rodriguez-Boulan E. (2002). The epithelial-specific adaptor AP1B mediates post-endocytic recycling to the basolateral membrane. Nat. Cell Biol. 4, 605–609. 10.1038/ncb827 12105417

[B44] Golachowska M. R. Hoekstra D. Van I. S. C. (2010). Recycling endosomes in apical plasma membrane domain formation and epithelial cell polarity. Trends Cell Biol. 20, 618–626. 10.1016/j.tcb.2010.08.004 20833047

[B45] Goldenring J. R. Smith J. Vaughan H. D. Cameron P. Hawkins W. Navarre J. (1996). Rab11 is an apically located small GTP-binding protein in epithelial tissues. Am. J. Physiol. 270, G515–G525. 10.1152/ajpgi.1996.270.3.G515 8638719

[B46] Gonzalez A. Garrido J. Vial J. D. (1981). Epidermal growth factor inhibits cytoskeleton-related changes in the surface of parietal cells. J. Cell Biol. 88, 108–114. 10.1083/jcb.88.1.108 7009622 PMC2111733

[B47] Gonzalez A. Nicovani S. Juica F. (1993). Apical secretion of Hepatitis B surface antigen from transfected Madin- Darby canine kidney cells. J. Biol. Chem. 268, 6662–6667. 10.1016/s0021-9258(18)53301-9 8454638

[B48] Gonzalez A. Rizzolo L. Rindler M. Adesnik M. Sabatini D. D. Gottlieb T. (1987). Nonpolarized secretion of truncated forms of the influenza hemagglutinin and the vesicular stomatitus virus G protein from MDCK cells. Proc. Natl. Acad. Sci. U. S. A. 84, 3738–3742. 10.1073/pnas.84.11.3738 3035552 PMC304951

[B49] Gonzalez A. Rodriguez-Boulan E. (2009). Clathrin and AP1B: Key roles in basolateral trafficking through trans-endosomal routes. FEBS Lett. 583, 3784–3795. 10.1016/j.febslet.2009.10.050 19854182 PMC4286365

[B50] Gore T. Matusek T. D'Angelo G. Giordano C. Tognacci T. Lavenant-Staccini L. (2021). The GTPase Rab8 differentially controls the long- and short-range activity of the Hedgehog morphogen gradient by regulating Hedgehog apico-basal distribution. Development 148, dev191791. 10.1242/dev.191791 33547132

[B51] Gottlieb T. A. Beaudry G. Rizzolo L. Colman A. Rindler M. Adesnik M. (1986). Secretion of endogenous and exogenous proteins from polarized MDCK cell monolayers. Proc. Natl. Acad. Sci. U. S. A. 83, 2100–2104. 10.1073/pnas.83.7.2100 3083413 PMC323238

[B52] Gradilla A. C. Gonzalez E. Seijo I. Andres G. Bischoff M. Gonzalez-Mendez L. (2014). Exosomes as Hedgehog carriers in cytoneme-mediated transport and secretion. Nat. Commun. 5, 5649. 10.1038/ncomms6649 25472772

[B53] Gradilla A. C. Guerrero I. (2013). Hedgehog on the move: A precise spatial control of hedgehog dispersion shapes the gradient. Curr. Opin. Genet. Dev. 23, 363–373. 10.1016/j.gde.2013.04.011 23747033

[B54] Gradilla A. C. Guerrero I. (2022). Hedgehog on track: Long-distant signal transport and transfer through direct cell-to-cell contact. Curr. Top. Dev. Biol. 150, 1–24. 10.1016/bs.ctdb.2022.03.002 35817500

[B55] Gravotta D. Carvajal-Gonzalez J. M. Mattera R. Deborde S. Banfelder J. R. Bonifacino J. S. (2012). The clathrin adaptor AP-1A mediates basolateral polarity. Dev. Cell 22, 811–823. 10.1016/j.devcel.2012.02.004 22516199 PMC3690600

[B56] Gravotta D. Deora A. Perret E. Oyanadel C. Soza A. Schreiner R. (2007). AP1B sorts basolateral proteins in recycling and biosynthetic routes of MDCK cells. Proc. Natl. Acad. Sci. U. S. A. 104, 1564–1569. 10.1073/pnas.0610700104 17244703 PMC1785260

[B57] Guerrero I. Kornberg T. B. (2014). Hedgehog and its circuitous journey from producing to target cells. Semin. Cell Dev. Biol. 33, 52–62. 10.1016/j.semcdb.2014.06.016 24994598

[B58] Guo X. Mattera R. Ren X. Chen Y. Retamal C. Gonzalez A. (2013). The adaptor protein-1 μ1B subunit expands the repertoire of basolateral sorting signal recognition in epithelial cells. Dev. Cell 27, 353–366. 10.1016/j.devcel.2013.10.006 24229647 PMC3992434

[B59] Hall E. T. Dillard M. E. Stewart D. P. Zhang Y. Wagner B. Levine R. M. (2021). Cytoneme delivery of Sonic Hedgehog from ligand-producing cells requires Myosin 10 and a Dispatched-BOC/CDON co-receptor complex. Elife 10, e61432. 10.7554/eLife.61432 33570491 PMC7968926

[B60] Hansen S. H. Casanova J. E. (1994). Gs alpha stimulates transcytosis and apical secretion in MDCK cells through cAMP and protein kinase A. J. Cell Biol. 126, 677–687. 10.1083/jcb.126.3.677 8045932 PMC2120136

[B61] Hauri H. P. Matter K. (1991). Protein traffic in intestinal epithelial cells. Semin. Cell Biol. 2, 355–364. 1813025

[B62] Hua W. Sheff D. Toomre D. Mellman I. (2006). Vectorial insertion of apical and basolateral membrane proteins in polarized epithelial cells revealed by quantitative 3D live cell imaging. J. Cell Biol. 172, 1035–1044. 10.1083/jcb.200512012 16567501 PMC2063761

[B63] Hubbard A. L. (1989). Endocytosis. Curr. Opin. Cell Biol. 1, 675–683. 10.1016/0955-0674(89)90033-1 2516741

[B64] Hunziker W. Harter C. Matter K. Mellman I. (1991). Basolateral sorting in MDCK cells requires a distinct cytoplasmic domain determinant. Cell 66, 907–920. 10.1016/0092-8674(91)90437-4 1909606

[B65] Hunziker W. Peters P. J. (1998). Rab17 localizes to recycling endosomes and regulates receptor-mediated transcytosis in epithelial cells. J. Biol. Chem. 273, 15734–15741. 10.1074/jbc.273.25.15734 9624171

[B66] Imjeti N. S. Lebreton S. Paladino S. De La Fuente E. Gonzalez A. Zurzolo C. (2011). N-Glycosylation instead of cholesterol mediates oligomerization and apical sorting of GPI-APs in FRT cells. Mol. Biol. Cell 22, 4621–4634. 10.1091/mbc.E11-04-0320 21998201 PMC3226479

[B67] Jaulin F. Xue X. Rodriguez-Boulan E. Kreitzer G. (2007). Polarization-dependent selective transport to the apical membrane by KIF5B in MDCK cells. Dev. Cell 13, 511–522. 10.1016/j.devcel.2007.08.001 17925227 PMC3712496

[B68] Jerdeva G. V. Tesar D. B. Huey-Tubman K. E. Ladinsky M. S. Fraser S. E. Bjorkman P. J. (2010). Comparison of FcRn- and pIgR-mediated transport in MDCK cells by fluorescence confocal microscopy. Traffic 11, 1205–1220. 10.1111/j.1600-0854.2010.01083.x 20525015 PMC2975666

[B69] Kohtz J. D. Lee H. Y. Gaiano N. Segal J. Ng E. Larson T. (2001). N-terminal fatty-acylation of sonic hedgehog enhances the induction of rodent ventral forebrain neurons. Development 128, 2351–2363. 10.1242/dev.128.12.2351 11493554

[B70] Kreitzer G. Marmorstein A. Okamoto P. Vallee R. Rodriguez-Boulan E. (2000). Kinesin and dynamin are required for post-Golgi transport of a plasma- membrane protein. Nat. Cell Biol. 2, 125–127. 10.1038/35000081 10655593

[B71] Kreitzer G. Schmoranzer J. Low S. H. Li X. Gan Y. Weimbs T. (2003). Three-dimensional analysis of post-Golgi carrier exocytosis in epithelial cells. Nat. Cell Biol. 5, 126–136. 10.1038/ncb917 12545172

[B72] Lahtinen U. Honsho M. Parton R. G. Simons K. Verkade P. (2003). Involvement of caveolin-2 in caveolar biogenesis in MDCK cells. FEBS Lett. 538, 85–88. 10.1016/s0014-5793(03)00135-2 12633858

[B73] Le Bivic A. Real F. X. Rodriguez-Boulan E. (1989). Vectorial targeting of apical and basolateral plasma membrane proteins in a human adenocarcinoma epithelial cell line. Proc. Natl. Acad. Sci. U. S. A. 86, 9313–9317. 10.1073/pnas.86.23.9313 2687880 PMC298485

[B74] Lebreton S. Paladino S. Liu D. Nitti M. Von Blume J. Pinton P. (2021). Calcium levels in the Golgi complex regulate clustering and apical sorting of GPI-APs in polarized epithelial cells. Proc. Natl. Acad. Sci. U. S. A. 118, e2014709118. 10.1073/pnas.2014709118 34389665 PMC8379914

[B75] Lebreton S. Paladino S. Zurzolo C. (2019). Clustering in the Golgi apparatus governs sorting and function of GPI-APs in polarized epithelial cells. FEBS Lett. 593, 2351–2365. 10.1002/1873-3468.13573 31400147

[B76] Lehmann G. L. Hanke-Gogokhia C. Hu Y. Bareja R. Salfati Z. Ginsberg M. (2020). Single-cell profiling reveals an endothelium-mediated immunomodulatory pathway in the eye choroid. J. Exp. Med. 217, e20190730. 10.1084/jem.20190730 32196081 PMC7971135

[B77] Li W. Wang L. Wierbowski B. M. Lu M. Dong F. Liu W. (2021). Structural insights into proteolytic activation of the human Dispatched1 transporter for Hedgehog morphogen release. Nat. Commun. 12, 6966. 10.1038/s41467-021-27257-w 34845226 PMC8630017

[B78] Lisanti M. P. Sargiacomo M. Graeve L. Saltiel A. R. Rodriguez-Boulan E. (1988). Polarized apical distribution of glycosyl-phosphatidylinositol-anchored proteins in a renal epithelial cell line. Proc. Natl. Acad. Sci. U. S. A. 85, 9557–9561. 10.1073/pnas.85.24.9557 2974157 PMC282796

[B79] Lock J. G. Stow J. L. (2005). Rab11 in recycling endosomes regulates the sorting and basolateral transport of E-cadherin. Mol. Biol. Cell 16, 1744–1755. 10.1091/mbc.e04-10-0867 15689490 PMC1073657

[B80] Long J. Tokhunts R. Old W. M. Houel S. Rodgriguez-Blanco J. Singh S. (2015). Identification of a family of fatty-acid-speciated sonic hedgehog proteins, whose members display differential biological properties. Cell Rep. 10, 1280–1287. 10.1016/j.celrep.2015.01.058 25732819 PMC4350664

[B81] Luton F. Hexham M. J. Zhang M. Mostov K. E. (2009). Identification of a cytoplasmic signal for apical transcytosis. Traffic 10, 1128–1142. 10.1111/j.1600-0854.2009.00941.x 19522755 PMC2920487

[B82] Machado M. V. Diehl A. M. (2017). Hedgehog signalling in liver pathophysiology. J. Hepatol. 68, 550–562. 10.1016/j.jhep.2017.10.017 29107151 PMC5957514

[B83] Maity T. Fuse N. Beachy P. A. (2005). Molecular mechanisms of Sonic hedgehog mutant effects in holoprosencephaly. Proc. Natl. Acad. Sci. U. S. A. 102, 17026–17031. 10.1073/pnas.0507848102 16282375 PMC1282174

[B84] Mao S. Shah A. S. Moninger T. O. Ostedgaard L. S. Lu L. Tang X. X. (2018). Motile cilia of human airway epithelia contain hedgehog signaling components that mediate noncanonical hedgehog signaling. Proc. Natl. Acad. Sci. U. S. A. 115, 1370–1375. 10.1073/pnas.1719177115 29358407 PMC5819449

[B85] Marzolo M. P. Bull P. Gonzalez A. (1997). Apical sorting of Hepatitis B surface antigen (HBsAg) is independent of N-glycosylation and glycosylphosphatidylinositol-anchored protein segregation. Proc. Natl. Acad. Sci. U. S. A. 94, 1834–1839. 10.1073/pnas.94.5.1834 9050865 PMC20003

[B86] Matusek T. Marcetteau J. Therond P. P. (2020). Functions of Wnt and Hedgehog-containing extracellular vesicles in development and disease. J. Cell Sci. 133, jcs209742. 10.1242/jcs.209742 32989011

[B87] Matusek T. Wendler F. Poles S. Pizette S. D'Angelo G. Furthauer M. (2014). The ESCRT machinery regulates the secretion and long-range activity of Hedgehog. Nature 516, 99–103. 10.1038/nature13847 25471885

[B88] Mccarthy R. A. Argraves W. S. (2003). Megalin and the neurodevelopmental biology of sonic hedgehog and retinol. J. Cell Sci. 116, 955–960. 10.1242/jcs.00313 12584240

[B89] Mellman I. Nelson W. J. (2008). Coordinated protein sorting, targeting and distribution in polarized cells. Nat. Rev. Mol. Cell Biol. 9, 833–845. 10.1038/nrm2525 18946473 PMC3369829

[B90] Miettinen H. M. Matter K. Hunziker W. Rose J. K. Mellman I. (1992). Fc receptor endocytosis is controlled by a cytoplasmic domain determinant that actively prevents coated pit localization. J. Cell Biol. 116, 875–888. 10.1083/jcb.116.4.875 1734021 PMC2289334

[B91] Oyanadel C. Holmes C. Pardo E. Retamal C. Shaughnessy R. Smith P. (2018). Galectin-8 induces partial epithelial-mesenchymal transition with invasive tumorigenic capabilities involving a FAK/EGFR/proteasome pathway in Madin-Darby canine kidney cells. Mol. Biol. Cell 29, 557–574. 10.1091/mbc.E16-05-0301 29298841 PMC6004583

[B92] Paladino S. Lebreton S. Zurzolo C. (2015). Trafficking and membrane organization of GPI-anchored proteins in health and diseases. Curr. Top. Membr. 75, 269–303. 10.1016/bs.ctm.2015.03.006 26015286

[B93] Paladino S. Pocard T. Catino M. A. Zurzolo C. (2006). GPI-anchored proteins are directly targeted to the apical surface in fully polarized MDCK cells. J. Cell Biol. 172, 1023–1034. 10.1083/jcb.200507116 16549497 PMC2063760

[B94] Paladino S. Sarnataro D. Pillich R. Tivodar S. Nitsch L. Zurzolo C. (2004). Protein oligomerization modulates raft partitioning and apical sorting of GPI-anchored proteins. J. Cell Biol. 167, 699–709. 10.1083/jcb.200407094 15557121 PMC2172584

[B95] Panakova D. Sprong H. Marois E. Thiele C. Eaton S. (2005). Lipoprotein particles are required for Hedgehog and Wingless signalling. Nature 435, 58–65. 10.1038/nature03504 15875013

[B96] Parchure A. Vyas N. Ferguson C. Parton R. G. Mayor S. (2015). Oligomerization and endocytosis of Hedgehog is necessary for its efficient exovesicular secretion. Mol. Biol. Cell 26, 4700–4717. 10.1091/mbc.E15-09-0671 26490120 PMC4678025

[B97] Parchure A. Vyas N. Mayor S. (2018). Wnt and hedgehog: Secretion of lipid-modified morphogens. Trends Cell Biol. 28, 157–170. 10.1016/j.tcb.2017.10.003 29132729 PMC6941938

[B98] Peng T. Frank D. B. Kadzik R. S. Morley M. P. Rathi K. S. Wang T. (2015). Hedgehog actively maintains adult lung quiescence and regulates repair and regeneration. Nature 526, 578–582. 10.1038/nature14984 26436454 PMC4713039

[B99] Pepinsky R. B. Zeng C. Wen D. Rayhorn P. Baker D. P. Williams K. P. (1998). Identification of a palmitic acid-modified form of human Sonic hedgehog. J. Biol. Chem. 273, 14037–14045. 10.1074/jbc.273.22.14037 9593755

[B100] Perez Bay A. E. Schreiner R. Benedicto I. Paz Marzolo M. Banfelder J. Weinstein A. M. (2016). The fast-recycling receptor Megalin defines the apical recycling pathway of epithelial cells. Nat. Commun. 7, 11550. 10.1038/ncomms11550 27180806 PMC4873671

[B101] Perez Bay A. E. Schreiner R. Benedicto I. Rodriguez-Boulan E. J. (2014). Galectin-4-mediated transcytosis of transferrin receptor. J. Cell Sci. 127, 4457–4469. 10.1242/jcs.153437 25179596 PMC4197088

[B102] Perez Bay A. E. Schreiner R. Mazzoni F. Carvajal-Gonzalez J. M. Gravotta D. Perret E. (2013). The kinesin KIF16B mediates apical transcytosis of transferrin receptor in AP-1B-deficient epithelia. EMBO J. 32, 2125–2139. 10.1038/emboj.2013.130 23749212 PMC3730227

[B103] Petrov K. Wierbowski B. M. Liu J. Salic A. (2020). Distinct cation gradients power cholesterol transport at different key points in the hedgehog signaling pathway. Dev. Cell 55, 314–327. 10.1016/j.devcel.2020.08.002 32860743 PMC7658045

[B104] Petrov K. Wierbowski B. M. Salic A. (2017). Sending and receiving hedgehog signals. Annu. Rev. Cell Dev. Biol. 33, 145–168. 10.1146/annurev-cellbio-100616-060847 28693388

[B105] Pizette S. Matusek T. Herpers B. Therond P. P. Rabouille C. (2021). Hherisomes, Hedgehog specialized recycling endosomes, are required for high level Hedgehog signaling and tissue growth. J. Cell Sci. 134, jcs258603. 10.1242/jcs.258603 34028543

[B106] Porter J. A. Young K. E. Beachy P. A. (1996). Cholesterol modification of hedgehog signaling proteins in animal development. Science 274, 255–259. 10.1126/science.274.5285.255 8824192

[B107] Ramalingam T. S. Detmer S. A. Martin W. L. Bjorkman P. J. (2002). IgG transcytosis and recycling by FcRn expressed in MDCK cells reveals ligand-induced redistribution. EMBO J. 21, 590–601. 10.1093/emboj/21.4.590 11847107 PMC125858

[B108] Rindler M. J. Traber M. G. (1988). A specific sorting signal is not required for the polarized secretion of newly synthesized proteins from cultured intestinal epithelial cells. J. Cell Biol. 107, 471–479. 10.1083/jcb.107.2.471 2458357 PMC2115219

[B109] Rodriguez-Boulan E. Macara I. G. (2014). Organization and execution of the epithelial polarity programme. Nat. Rev. Mol. Cell Biol. 15, 225–242. 10.1038/nrm3775 24651541 PMC4211427

[B110] Roy S. Hsiung F. Kornberg T. B. (2011). Specificity of Drosophila cytonemes for distinct signaling pathways. Science 332, 354–358. 10.1126/science.1198949 21493861 PMC3109072

[B111] Sargiacomo M. Lisanti M. Graeve L. Le Bivic A. Rodriguez-Boulan E. (1989). Integral and peripheral protein composition of the apical and basolateral membrane domains in MDCK cells. J. Membr. Biol. 107, 277–286. 10.1007/BF01871942 2716048

[B112] Sarnataro D. Paladino S. Campana V. Grassi J. Nitsch L. Zurzolo C. (2002). PrPC is sorted to the basolateral membrane of epithelial cells independently of its association with rafts. Traffic 3, 810–821. 10.1034/j.1600-0854.2002.31106.x 12383347

[B113] Sbrogna J. L. Barresi M. J. Karlstrom R. O. (2003). Multiple roles for Hedgehog signaling in zebrafish pituitary development. Dev. Biol. 254, 19–35. 10.1016/s0012-1606(02)00027-1 12606279

[B114] Scheiffele P. Peranen J. Simons K. (1995). N-glycans as apical sorting signals in epithelial cells. Nature 378, 96–98. 10.1038/378096a0 7477300

[B115] Schonteich E. Wilson G. M. Burden J. Hopkins C. R. Anderson K. Goldenring J. R. (2008). The Rip11/Rab11-FIP5 and kinesin II complex regulates endocytic protein recycling. J. Cell Sci. 121, 3824–3833. 10.1242/jcs.032441 18957512 PMC4365997

[B116] Sezgin E. Levental I. Mayor S. Eggeling C. (2017). The mystery of membrane organization: Composition, regulation and roles of lipid rafts. Nat. Rev. Mol. Cell Biol. 18, 361–374. 10.1038/nrm.2017.16 28356571 PMC5500228

[B117] Sheff D. R. Daro E. A. Hull M. Mellman I. (1999). The receptor recycling pathway contains two distinct populations of early endosomes with different sorting functions. J. Cell Biol. 145, 123–139. 10.1083/jcb.145.1.123 10189373 PMC2148223

[B118] Shyer A. E. Huycke T. R. Lee C. Mahadevan L. Tabin C. J. (2015). Bending gradients: How the intestinal stem cell gets its home. Cell 161, 569–580. 10.1016/j.cell.2015.03.041 25865482 PMC4409931

[B119] Simons K. Ikonen E. (1997). Functional rafts in cell membranes. Nature 387, 569–572. 10.1038/42408 9177342

[B120] Sonnichsen B. De Renzis S. Nielsen E. Rietdorf J. Zerial M. (2000). Distinct membrane domains on endosomes in the recycling pathway visualized by multicolor imaging of Rab4, Rab5, and Rab11. J. Cell Biol. 149, 901–914. 10.1083/jcb.149.4.901 10811830 PMC2174575

[B121] Stewart D. P. Marada S. Bodeen W. J. Truong A. Sakurada S. M. Pandit T. (2018). Cleavage activates dispatched for Sonic Hedgehog ligand release. Elife 7, e31678. 10.7554/eLife.31678 29359685 PMC5811216

[B122] Striz A. C. Stephan A. P. Lopez-Coral A. Tuma P. L. (2018). Rab17 regulates apical delivery of hepatic transcytotic vesicles. Mol. Biol. Cell 29, 2887–2897. 10.1091/mbc.E18-07-0433 30256711 PMC6249867

[B123] Tanaka Y. Okada Y. Hirokawa N. (2005). FGF-induced vesicular release of Sonic hedgehog and retinoic acid in leftward nodal flow is critical for left-right determination. Nature 435, 172–177. 10.1038/nature03494 15889083

[B124] Therond P. P. (2012). Release and transportation of Hedgehog molecules. Curr. Opin. Cell Biol. 24, 173–180. 10.1016/j.ceb.2012.02.001 22366329

[B125] Thompson A. Nessler R. Wisco D. Anderson E. Winckler B. Sheff D. (2007). Recycling endosomes of polarized epithelial cells actively sort apical and basolateral cargos into separate subdomains. Mol. Biol. Cell 18, 2687–2697. 10.1091/mbc.e05-09-0873 17494872 PMC1924834

[B126] Thuenauer R. Hsu Y. C. Carvajal-Gonzalez J. M. Deborde S. Chuang J. Z. Romer W. (2014). Four-dimensional live imaging of apical biosynthetic trafficking reveals a post-Golgi sorting role of apical endosomal intermediates. Proc. Natl. Acad. Sci. U. S. A. 111, 4127–4132. 10.1073/pnas.1304168111 24591614 PMC3964106

[B127] Tukachinsky H. Kuzmickas R. P. Jao C. Y. Liu J. Salic A. (2012). Dispatched and scube mediate the efficient secretion of the cholesterol-modified hedgehog ligand. Cell Rep. 2, 308–320. 10.1016/j.celrep.2012.07.010 22902404 PMC3682496

[B128] Tuma P. L. Hubbard A. L. (2003). Transcytosis: Crossing cellular barriers. Physiol. Rev. 83, 871–932. 10.1152/physrev.00001.2003 12843411

[B129] Vial J. D. Garrido J. (1976). Actin-like filaments amd membrane rearrangement in oxyntic cells. Proc. Natl. Acad. Sci. U. S. A. 73, 4032–4036. 10.1073/pnas.73.11.4032 1069289 PMC431315

[B130] Vyas N. Goswami D. Manonmani A. Sharma P. Ranganath H. A. Vijayraghavan K. (2008). Nanoscale organization of hedgehog is essential for long-range signaling. Cell 133, 1214–1227. 10.1016/j.cell.2008.05.026 18585355

[B131] Vyas N. Walvekar A. Tate D. Lakshmanan V. Bansal D. Lo Cicero A. (2014). Vertebrate Hedgehog is secreted on two types of extracellular vesicles with different signaling properties. Sci. Rep. 4, 7357. 10.1038/srep07357 25483805 PMC4258658

[B132] Walton K. D. Gumucio D. L. (2021). Hedgehog signaling in intestinal development and homeostasis. Annu. Rev. Physiol. 83, 359–380. 10.1146/annurev-physiol-031620-094324 33035430 PMC10278198

[B133] Wang E. Brown P. S. Aroeti B. Chapin S. J. Mostov K. E. Dunn K. W. (2000a). Apical and basolateral endocytic pathways of MDCK cells meet in acidic common endosomes distinct from a nearly-neutral apical recycling endosome. Traffic 1, 480–493. 10.1034/j.1600-0854.2000.010606.x 11208134

[B134] Wang Q. Asarnow D. E. Ding K. Mann R. K. Hatakeyama J. Zhang Y. (2021). Dispatched uses Na(+) flux to power release of lipid-modified Hedgehog. Nature 599, 320–324. 10.1038/s41586-021-03996-0 34707294 PMC8785653

[B135] Wang X. Kumar R. Navarre J. Casanova J. E. Goldenring J. R. (2000b). Regulation of vesicle trafficking in madin-darby canine kidney cells by Rab11a and Rab25. J. Biol. Chem. 275, 29138–29146. 10.1074/jbc.M004410200 10869360

[B136] Weisz O. A. Rodriguez-Boulan E. (2009). Apical trafficking in epithelial cells: Signals, clusters and motors. J. Cell Sci. 122, 4253–4266. 10.1242/jcs.032615 19923269 PMC2779128

[B137] Williams K. P. Rayhorn P. Chi-Rosso G. Garber E. A. Strauch K. L. Horan G. S. (1999). Functional antagonists of sonic hedgehog reveal the importance of the N terminus for activity. J. Cell Sci. 112, 4405–4414. 10.1242/jcs.112.23.4405 10564658

[B138] Zacchi P. Stenmark H. Parton R. G. Orioli D. Lim F. Giner A. (1998). Rab17 regulates membrane trafficking through apical recycling endosomes in polarized epithelial cells. J. Cell Biol. 140, 1039–1053. 10.1083/jcb.140.5.1039 9490718 PMC2132691

[B139] Zavros Y. Orr M. A. Xiao C. Malinowska D. H. (2008). Sonic hedgehog is associated with H+-K+-ATPase-containing membranes in gastric parietal cells and secreted with histamine stimulation. Am. J. Physiol. Gastrointest. Liver Physiol. 295, G99–G111. 10.1152/ajpgi.00389.2007 18483183 PMC5243217

[B140] Zavros Y. (2008). The adventures of sonic hedgehog in development and repair. IV. Sonic hedgehog processing, secretion, and function in the stomach. Am. J. Physiol. Gastrointest. Liver Physiol. 294, G1105–G1108. 10.1152/ajpgi.00031.2008 18308861

[B141] Zeng X. Goetz J. A. Suber L. M. Scott W. J. JR. Schreiner C. M. Robbins D. J. (2001). A freely diffusible form of Sonic hedgehog mediates long-range signalling. Nature 411, 716–720. 10.1038/35079648 11395778

[B142] Zurzolo C. Le Bivic A. Quaroni A. Nitsch L. Rodriguez-Boulan E. (1992). Modulation of transcytotic and direct targeting pathways in a polarized thyroid cell line. EMBO J. 11, 2337–2344. 10.1002/j.1460-2075.1992.tb05293.x 1350978 PMC556701

[B143] Zurzolo C. Simons K. (2016). Glycosylphosphatidylinositol-anchored proteins: Membrane organization and transport. Biochim. Biophys. Acta 1858, 632–639. 10.1016/j.bbamem.2015.12.018 26706096

